# Functionalized Separators Boosting Electrochemical Performances for Lithium Batteries

**DOI:** 10.1007/s40820-024-01596-x

**Published:** 2025-02-05

**Authors:** Zixin Fan, Xiaoyu Chen, Jingjing Shi, Hui Nie, Xiaoming Zhang, Xingping Zhou, Xiaolin Xie, Zhigang Xue

**Affiliations:** 1 Key Laboratory of Material Chemistry for Energy Conversion and Storage, Ministry of Education, Hubei Key Laboratory of Material Chemistry and Service Failure, School of Chemistry and Chemical Engineering, Huazhong University of Science and Technology, Wuhan, 430074 People’s Republic of China; 2 Shenzhen Senior Technology Material Co. Ltd., Shenzhen, 518000 People’s Republic of China

**Keywords:** Separators, Polymer electrolytes, Lithium batteries, Electrochemical performances, Functionalization

## Abstract

The growing demands for energy storage systems, electric vehicles, and portable electronics have significantly pushed forward the need for safe and reliable lithium batteries. It is essential to design functional separators with improved mechanical and electrochemical characteristics. This review covers the improved mechanical and electrochemical performances as well as the advancements made in the design of separators utilizing a variety of techniques. In terms of electrolyte wettability and adhesion of the coating materials, we provide an overview of the current status of research on coated separators, in situ modified separators, and grafting modified separators, and elaborate additional performance parameters of interest. The characteristics of inorganics coated separators, organic framework coated separators and inorganic–organic coated separators from different fabrication methods are compared. Future directions regarding new modified materials, manufacturing process, quantitative analysis of adhesion and so on are proposed toward next-generation advanced lithium batteries.

**Graphical abstract:**

## Highlights

- The commonly used modification methods for separator of lithium batteries are summarized, which include surface coating, in situ modification and grafting modification.
- The adhesion of coating materials with the separators and wettability of the modified separators prepared from the three methods are compared.
- The challenges and future directions of separator modification are provided.

## Graphical abstract

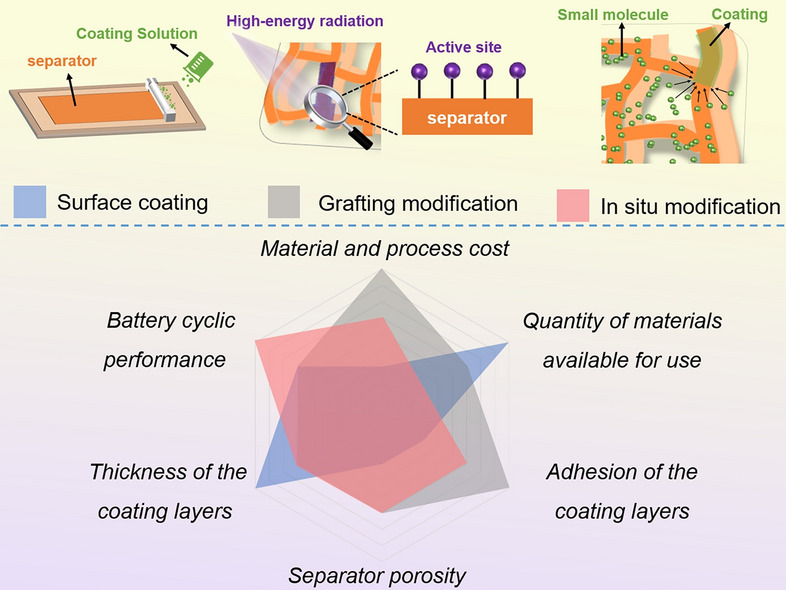

## Introduction

For a more sustainable society, it is now critical to develop renewable, clean energy as well as effective energy conversion and storage systems. Because of their high energy density and low redox potential, lithium batteries as one type of Secondary batteries are widely utilized in energy storage systems [1]. Lithium-ion batteries were first developed and commercialized by Sony in 1991 [2]. However, the energy density of the state-of-the-art lithium-ion batteries, which use graphite anodes and insertion compound cathodes, has achieved its maximum (~ 150 Wh kg^−1^) [3]. Theoretically, lithium metal batteries offer a more appealing and higher energy density. The unstable solid electrolyte interphase (SEI) of the lithium anodes and severe lithium dendrite growth readily lead to short circuits and rapid, uncontrolled discharge of the battery, causing a series of safety issues [4]. Lithium-sulfide batteries and lithium-oxygen batteries also form multiple lithium sulfides and lithium oxide intermediate products during the cycling process, which seriously passivates the lithium anodes, resulting in a decline in cycle efficiency and battery performance [5]. In addition, the overheating of lithium batteries causes fire and explosion accidents. For instance, fast charging and rapid heating might cause the liquid electrolyte to burn, which is extremely dangerous for human life and health. Although extending the energy density and cycle life of lithium batteries is a popular objective, cost and safety are also receiving a lot of attention. Improving the physical and chemical characteristics of battery components proves to be a successful and efficient route.

**Fig. 1:**
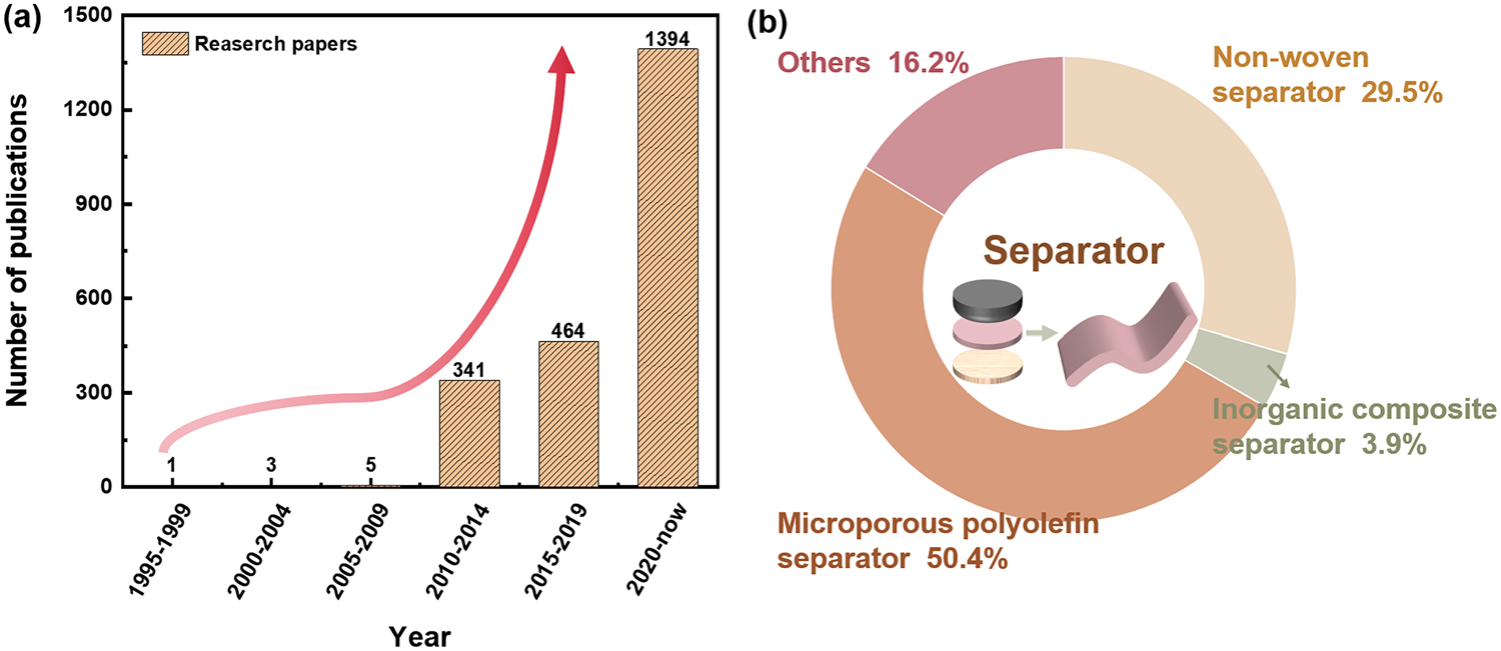
**a** Research papers from September 1995 to September 2024 with the keywords of “lithium battery separator” “functionalized” or “lithium battery separator” “modified” searching on Clarivate. **b** Papers on various separators in battery systems. Figures were prepared in September 2024

Until now, tremendous advances have been made in optimization of electrodes for improved performance of lithium metal batteries, including the creation of artificial SEI, modulation of the anode’s three-dimensional structure, and control of the cathode’s surface structure. Recently, there has been a greater focus on the role separators play in regulating ion transport and, consequently, the behavior of lithium deposition. Uniform and fast transport of Li^+^ through separators is essential to reduce the risk of local overcharge and growth of lithium dendrite [6]. Ion flux distribution is greatly influenced by the chemical composition and pore structure of the separators [7], and micro-channels within the separators facilitate the migration of Li^+^. It has been demonstrated that separators can significantly increase the cycle life of lithium batteries. Lithium battery separators have advanced quickly since the turn of the twenty-first century due to the widespread use of lithium batteries. Figure 1 illustrates the increase in pertinent research publications as well as papers on different separators used in battery systems.

At present, there are reviews on different types of separators [1, 8], their fabrication technologies [1, 9], safety issues [9] and functional design [1], but the impact of various modification techniques on the performance of functional separators has not been particularly investigated. Thus, this paper aims to present a comprehensive and concise review of the latest development of functional separators based on different modification methods. We will explore the influence of different modified separators on the battery performance from the perspective of coating, in-situ, and grafting functionalization methods. Emphasis is generally placed on electrolyte wettability and adhesion of the coating materials of the functional separators, and additional performance parameters of interest will be elaborated. We hope to provide guidance for the future design of new functional separators.

## Basic of Separators

The separator, one of the most critical components of lithium battery, is placed between the positive and negative electrodes. It plays the following important roles: (1) prevent contact between the positive electrode and negative electrode and thus avoid short circuits of the battery; (2) provide channels for rapid Li^+^ transport [1, 8, 10–12]. The currently used separators are usually classified into three types, microporous polyolefin separators, non-woven separators, and inorganic composite separators [13]. Microporous polyolefin separators, such as polyethylene (PE), polypropylene (PP) separators, and three-layer composite separators composed of PE and PP, are the mostly used commercialized separators due to their high mechanical properties and excellent dimensional stability. The non-woven separator is composed of randomly arranged fibers [14]. Cellulose [15, 16], polyvinylidene fluoride (PVDF) [17], aramid nanofiber (ANF) [18], poly(acrylonitrile) (PAN) [19, 20], polyimide (PI) [21, 22], polyetherimide (PEI) [23], poly(ether ether ketone) (PEEK) [24], polybenzimidazole (PBI) [25] and other materials [26] have been exploited to form fibrous membranes through electrospinning and melt-blowing. By integrating inorganic particles into a polymer matrix, the formed inorganic composite separator combines the characteristics of both materials. The relevant data of the commonly used separators that have been industrialized is shown in Table 1.

**Table 1:** Summary of characteristics of some representative commonly used separators that have been industrialized

Separator	Brand	Thickness (μm)	Porosity (%)	Pore size (μm)	Electrolyte contact angle	Ionic conductivity (mS cm^–1^) at RT
PP	Celgard 2400	25	41	0.043	–	–
PP	Celgard 2500	25	55	0.091	> 90°	–
PE	Hipore	16	41	–	–	0.61
PE	Senior	9	43	0.045	> 50°	1.04
Cellulose	Cellulion	20	63	2.44	–	0.90
PI		20	91	–	10°	1.71

**Fig. 2:**
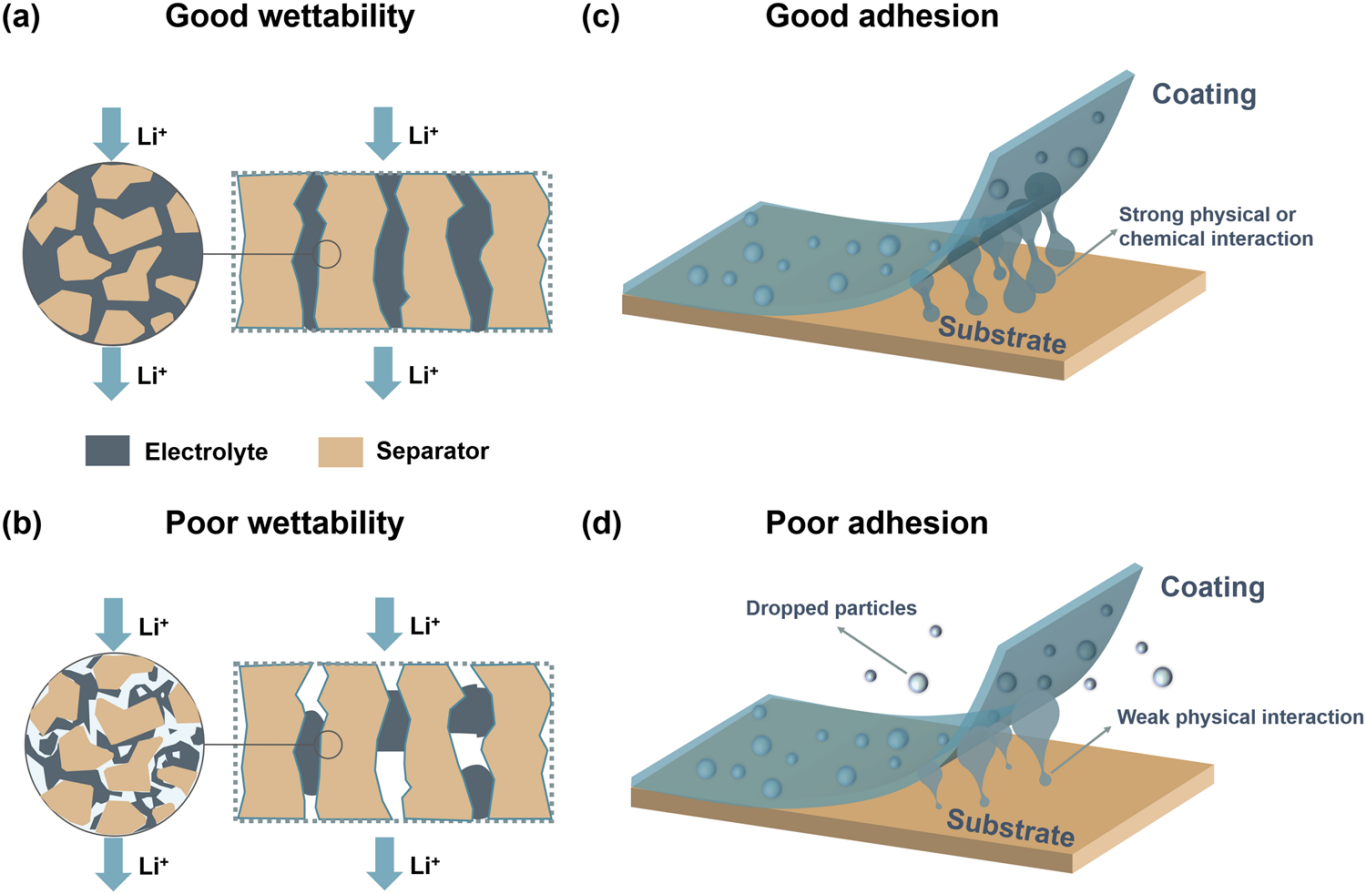
**a**,** b** Schematic illustration of the influence of separator wettability toward electrolyte on Li^+^ transportation. **c**, **d** Schematic illustration of the adhesion between the matrix film and the modified layer

One aspect that affects the uniformity of ions transport through the separator is wetting ability [6]. Separators must possess high porosity and good wettability toward liquid electrolytes (LEs) to increase their uptake and retention rate [27]. The separator with good wettability is conducive to rapid electrolyte permeation, to fill the pores of the separator and establish an efficient and fast lithium-ion transport channel [14, 28–32], as shown in Fig. 2a, which minimizes the ion transfer resistance, enhances the ionic conductivity and suppresses the lithium dendrites growth [33, 34]. Incomplete filling of the pore space blocks the transport path of the pore network and decreases the lithium-ion transport capacity of the separator, as shown in Fig. 2b [6]. The interlayer or functional separator with high porosity and excellent wettability toward LEs regulates the transport behavior of Li^+^ to the greatest extent so that lithium-ion is uniformly deposited on the surface of the lithium metal anode, thus inhibiting the lithium dendrites growth and realizing homogeneous ionic transport for long-cycling lithium batteries [7, 35].

**Fig. 3:**
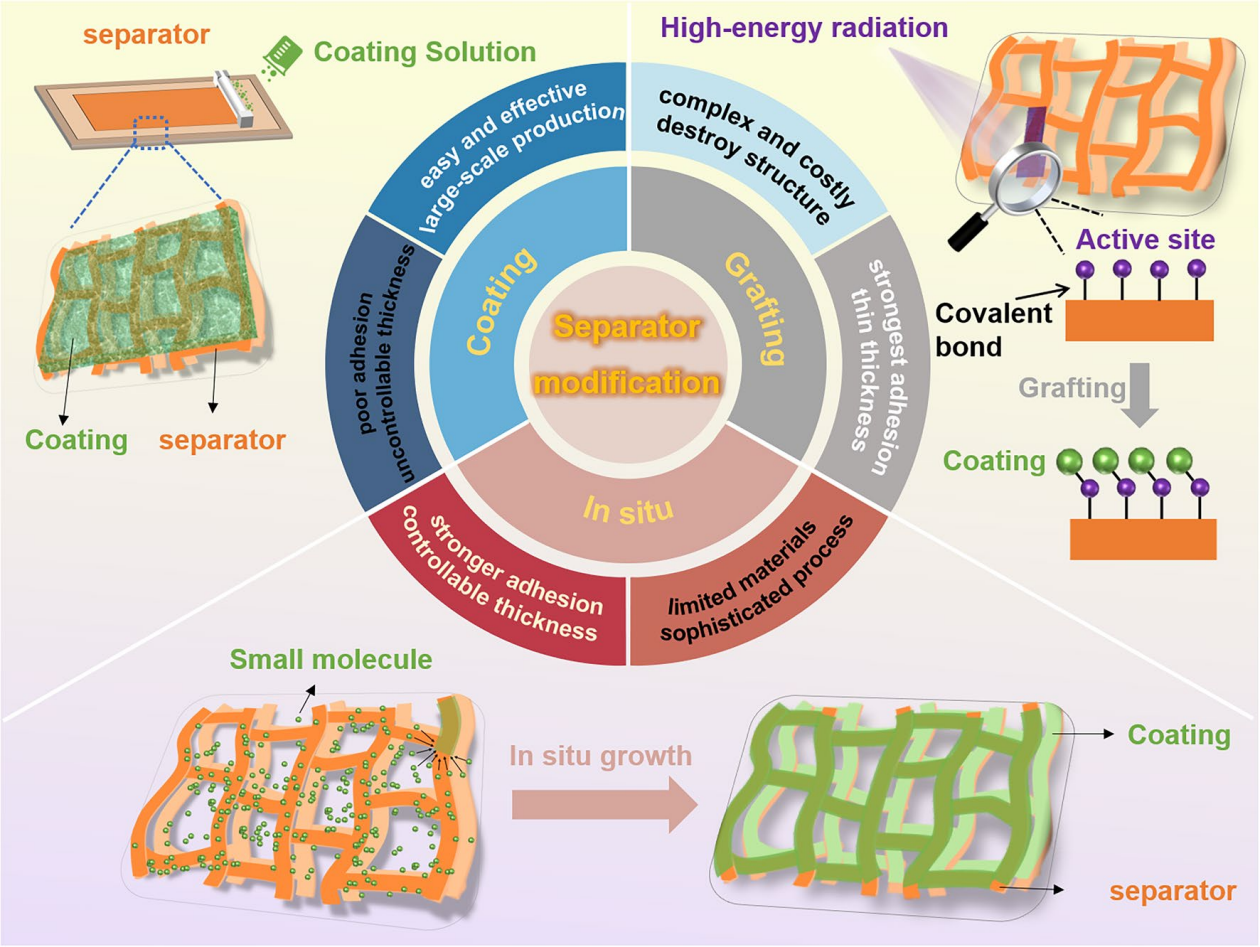
Schematic showing three commonly used modification methods for separators of lithium batteries and their advantages and disadvantages

However, the commercial polyolefin separators contain abundant nonpolar –CH_2_ and –CH_3_ groups, resulting in poor affinity for electrolytes [13, 36]. Inorganic composite separators have the problem of uneven mixing of inorganic particles with matrix, which leads to low structural integrity, reduced porosity and ionic conductivity [37–39]. Numerous nonwoven-based separators have excellent wettability and have been used in lithium batteries due to their high porosity and specific surface area, but their other properties such as mechanical strength still need to be optimized [11, 14]. Based on this, the researchers modify the separator using a variety of materials, such as inorganic nanoparticles and polymers with abundant polar groups [13, 40]. The modified separator exhibits good wettability with LEs due to its polar structures and porous structures. For surface-modified separators, the adhesion between the matrix film and the modified layer is particularly important (Fig. 2c, d), which refers to the dimension stability of the separators. The separators modified by coating, in situ and grafting functionalization methods can not only improve the wettability but also improve the adhesion step by step. The differences between the three functionalization methods are also compared, as shown in Fig. 3. Except for electrolyte wetting ability and adhesive properties of coating materials, chemical stability, thickness, porosity, pore size, mechanical strength, thermal stability and electrochemical stability of the separators are also important parameters affecting the performance of batteries [41–45].

## Coated Separators

The coating can facilely introduce functional layers, which have high polarity and good compatibility with LEs on the surface of separators. The commonly used methods include blast-coating, vacuum filtration and dip-coating. These methods enable large-scale and low-cost production of surface functionalized membranes.

### Inorganics–Coated Separators

Inorganic ceramics, carbon-based nanomaterials, metal nitrides and metal sulfides are largely exploited for surface coating of separators. These inorganic modification layers always feature excellent wettability for LEs due to (a) increased surface roughness and/or surface energy and (b) uniform size at the nanoscale [46]. Meanwhile, the inorganic coated layers also improve the thermal stability of the separators to a certain extent. The characteristics and electrochemical performance of inorganic-coated separators and their assembled lithium batteries are summarized in Table 2.

**Table 2:** Summary of characteristics and electrochemical performance of the inorganic-coated separators

Separator	Coating thickness (μm)	Porosity^a^	Coating mass fraction	Electrolyte contact angle	Electrolyte uptake	Ionic conductivity (mS cm^–1^) at RT	Cyclic performance	References
*Inorganic ceramics*								
PE/WCDA-SiO_2_	–^b^	–	–	–	310%	0.32	73.3% after 100 cycles at 0.5C (original) 81.6% after 100 cycles at 0.5C (modified)	[47]
PP/SiO_2_	6	–	20%	< 10°	–	–	–	[48]
PE/SiO_2_	10	–	–	–	–	0.61	73% after 200 cycles at 0.5C (original) 77% after 200 cycles at 0.5C (modified)	[49]
PP/SiO_2_/Al_2_O_3_	–	–	–	–	120%	0.78	–	[50]
PP/SiO_2_/Al_2_O_3_	–	–	–	21.7° (H_2_O)	–	–	64.7% after 400 cycles at 1C (original) 90.9% after 400 cycles at 1C (modified)	[51]
PIC/Al_2_O_3_	–	–	–	0°	–	1.64	–	[52]
PP/SiO_2_	6		–	0°	346%	0.63	80.6% after 100 cycles at 1C (original) 87.4% after 100 cycles at 1C (modified)	[53]
PP/Al(OH)_3_	–	84%	–	–	127%	1.00	–	[54]
PE/AlOOH	1.15	–	–	–	–	6.56	94.6% after 100 cycles at 1C (original) 96.3% after 100 cycles at 1C (modified)	[55]
PE/N-SiO_2_	2.5		–	51.3° (H_2_O)	195%	0.81	88.5% after 200 cycles at 0.1C (modified)	[56]
PVDF/SiO_2_/Al_2_O_3_	0.2	68.1%	–	18.9°	468%	2.24	71.6% after 100 cycles at 0.2C (PP) 86.4% after 100 cycles at 0.2C (modified)	[57]
PVDF/Al_2_O_3_/SiO_2_	–	61.8%	–	18.3°	366%	2.06	78.4% after 100 cycles at 0.5C (PE) 81.3% after 100 cycles at 0.5C (modified)	[58]
PI/SiO_2_/Al_2_O_3_	5	89.8%	–	–	519%	2.92	95.58% after 250 cycles at 5C (PP) 98.76% after 250 cycles at 5C (modified)	[59]
PE/Al_2_O_3_/HNTs	3	–	–	9°	281%	0.67	84.4% after 200 cycles at 0.2C (original) 83.4% after 200 cycles at 0.2C (modified)	[60]
PE/Al_2_O_3_	3	31.7%	–	4.21°	170%	1.16	82.2% after 100 cycles at 0.5C (original) 91.5% after 100 cycles at 0.5C (modified)	[61]
PP/Al_2_O_3_/TiO_2_	10	–	–	15°	–	–	–	[62]
PET/LLZO	–	50%	87%	19.6°	–	2.80	–	[63]
PVA/ZrO_2_	–	73%	–	21.6°	420%	2.19	90% after 200 cycles at 0.2C (PE) 97% after 200 cycles at 0.2C (modified)	[64]
PE/SnO_2_	2.7	–	–	46.5° (H_2_O)	119%	0.72	–	[65]
PP/Zeolite 4A	10	58%	–	0°	270%	2.25	83.4% after 100 cycles at 0.5C (original) 96.2% after 100 cycles at 0.5C (modified)	[66]
PE/Si–O-Al_2_O_3_	–	–	–	–	269%	0.68	16.0% after 400 cycles at 0.5C (original) 54.1% after 1000 cycles at 0.5C (modified)	[67]
*Carbon-based nanomaterials*								
PP/DLC	2.9	–	–	29°	–	0.69	71% after 1000 cycles at 5C (modified)	[68]
PP/LNS/CB	3.5	–	–	0°	–	0.59	86% after 500 cycles at 1C (modified)	[69]
PP/GO	–	–	–	15° (H_2_O)	–	0.60	–	[70]
PP/SFGS	20	–	–	54.4° (H_2_O)	–	–	–	[71]
ANF/rGOF	15 ~ 20	–	–	–	–	–	89.07% after 2000 cycles at 50 C (modified)	[72]
PP/rGO/BNNSs	9.5	–	–	10.97°	–	–	92.3% after 500 cycles at 4 C (modified)	[73]
PP/rGO/Li-Al-LDH	12	–	–	0°	233%	0.74	95.76% after 250 cycles at 1C (modified)	[74]
PP/Fe_3_N@NG	–	–	–	5.23°	116%	0.70	–	[75]
PP/CFs	15	–	–	0°	–	–	48.7% after 500 cycles at 0.5C (original) 64.5% after 500 cycles at 0.5C (modified)	[76]
ANF/ NiFe_2_O_4_-OCNT	1.6	–	–	17.6°	253%	2.21	72% after 1000 cycles at 1C (modified)	[77]
*Others*								
PP/CuS/graphene	20	–	–	0°	–	–	62.1% after 200 cycles at 1C (modified)	[78]
PP/h-BN	2	–	–	15.7°	–	–	–	[79]
PI/hBN	3	44.5%	–	7.2°	63%	–	73% after 300 cycles at 1C (PP) 79% after 300 cycles at 1C (modified)	[80]

^a^ “Porosity” is the entire separator’s, instead of the coating’s. ^b^ “–” means not mentioned

#### Inorganic Ceramics

Inorganic ceramic materials generally refer to a class of inorganic non-metallic materials made of natural or synthetic compounds by forming and sintering at high temperature. With the help of binder, SiO_2_ [48–51], Al_2_O_3_ [52, 61, 81] and other derivatives [54, 55, 63] are commonly used inorganic ceramics for surface coated separators with improved electrolyte uptake and ionic conductivity [82].

**Fig. 4:**
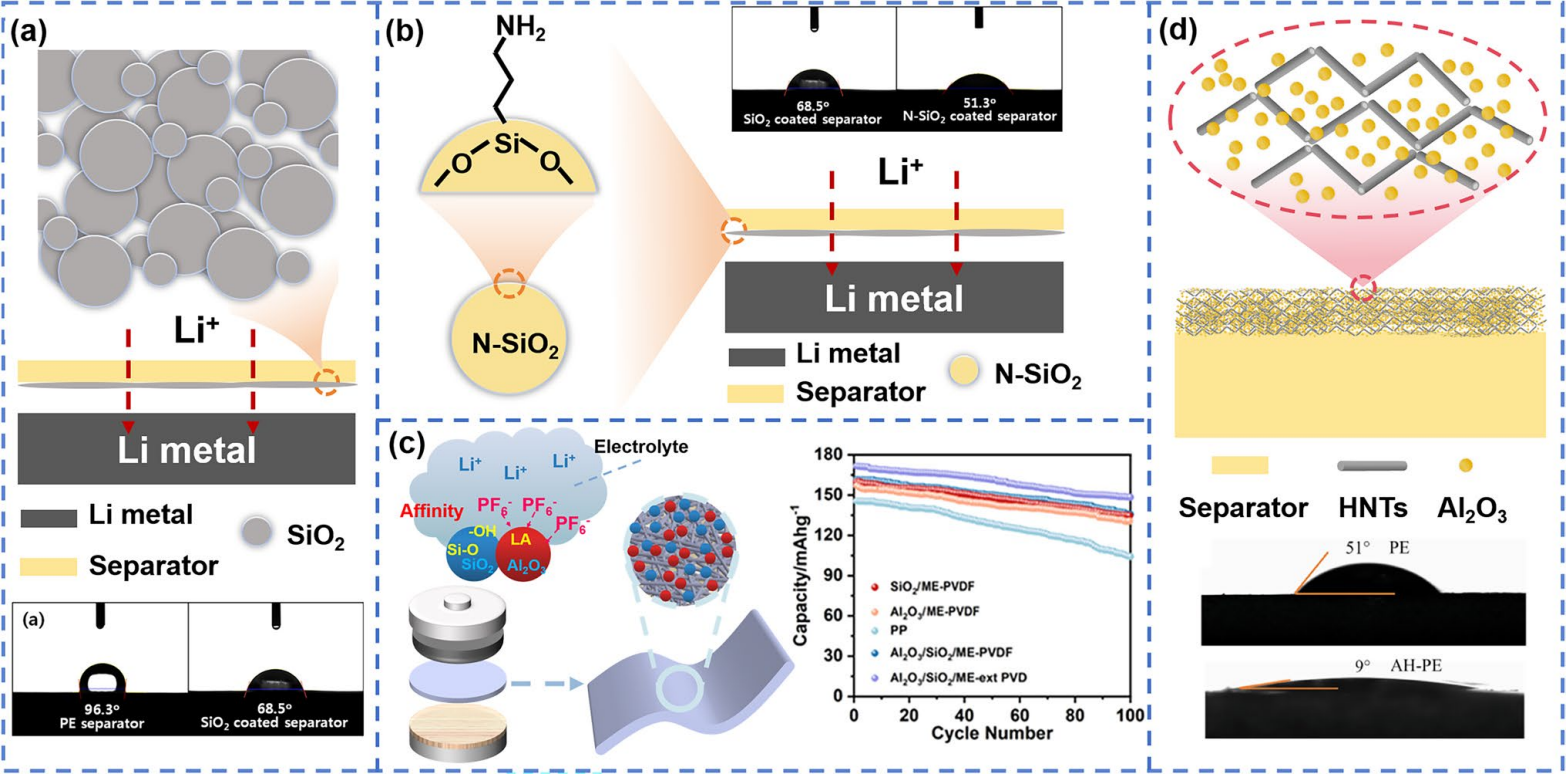
**a** Schematic illustration of the SiO_2_ coated PE separator and their contact angles [56]. Copyright 2017, Elsevier. **b** Schematic illustration of amino-functionalized SiO_2_ (N-SiO_2_) particles coated separator and their contact angle [56]. Copyright 2017, Elsevier. **c** Schematic illustrations of SiO_2_ and Al_2_O_3_ coated PVDF separator [57]. Copyright 2023, American Chemical Society. **d** Schematic illustrations of the AH/PE separator and their wetting behavior [60]. Copyright 2023, Elsevier

For example, the surface coating of SiO_2_ can be achieved either by uniformly coating slurry containing the commercially available SiO_2_ on the separator surface [49], or directly hydrolyzing tetraethyl orthosilicate to deposit SiO_2_ on the separator [48] (Fig. 4a). Through the latter method, the coating thickness (lower than the directly coated SiO_2_ [49]) and specific surface area of the ceramic coating layer could be easily controlled by adjusting the solution concentration and deposition time, and a contact angle of less than 10° was achieved. The coated separators showed significant electrolyte uptake and improved battery performance. The amino-functionalized SiO_2_ (N-SiO_2_) coating layer with a thin coating thickness of 2.5 μm (compared to 6 μm in [48]) was fabricated on PE separator, which exhibited higher ionic conductivity of 0.81 mS cm^–1^ (compared to 0.61 mS cm^–1^ in [49]), and excellent electrolyte wettability. The contact angle decreased from 96.3° to 51.3° (Fig. 4b). Interestingly, N-SiO_2_ particles also played a role as a HF scavenger, which effectively inhibited electrolyte decomposition at high temperatures and realized stable cycling of battery (88.5% capacity retention after 200 cycles at 0.1C) [56]. Mixed coating of two different ceramic materials on the polyolefin separator can further improve the electrolyte wettability [51, 60]. Coating slurry with mixture of SiO_2_ and Al_2_O_3_ on the PVDF fibrous membrane led to separators with excellent electrolyte uptake (more than 350%) and remarkable ionic conductivity (more than 2 mS cm^–1^), as shown in Fig. 4c. Impressively, capacity retention of more than 80% after 100 cycles could be achieved for the assembled cells [57, 58]. Figure 4d showed separators coated with both Al_2_O_3_ and halloysite nanotubes (HNTs) had better wettability than those coated with Al_2_O_3_ alone. The HNTs with hollow structure not only reduced the weight increase, but also provided fast ion transport channels for the modified separators. Meanwhile, the positive charge on the surface of HNT could adsorb anions and promote the Li^+^ movement. The assembled cells achieved capacity retention rate of 83.4% after 200 cycles at 0.2C. This demonstrated the more abundant polar functional groups in the coating layer, the better affinity with polar electrolytes. Fortunately, the multi-material coating didn’t significantly increase the separator thickness and presented a loosely stacked porous structure [60]. Currently, ceramic-coated separators based on SiO_2_ and Al_2_O_3_ are widely used in commercial lithium batteries.

Generally, inorganic ceramics-coated separators suffer from some shortcomings such as uneven separator thickness, blockage of separator pores, interface incompatibility, and brittleness. In addition, the binders melt or decompose with the rising of temperatures, leading to the detachment of the coating materials. Zhang et al. [61] fabricated Al_2_O_3_ ceramic-coated separators using an organic–inorganic composite binder composed of polyvinyl alcohol-phosphate inorganic (PVA-PIB). The organic binder has poor thermal resistance and easily melts at high temperatures, leading to the shrinkage of the separator. The inorganic binder has poor compatibility with the polyolefin matrix, resulting in poor adhesion. Thus, the PVA-PIB organic–inorganic composite binder overcame the above shortcomings simultaneously, and the assembled cells had improved electrochemical performance. Luo et al. [83] prepared a raspberry microparticle polymer (RMP) binder with a soft inner core and hard outer sphere. With this binder, the Al_2_O_3_ could be firmly attached to the PE separator. Meanwhile, the pore blockage was avoided. Na et al. [84] activated the surface of polyolefin separator by ultraviolet ozone (UVO) plasma treatment. Then through silane hybridization, SiO_2_ nanoparticles were coated on the separator surface. This work demonstrated the fabrication of SiO_2_-modified separator without polymer binder for the first time. In general, the ceramic and the binder content of slurry for coating needs to be optimized to prevent the plugging of separator pores. Also, molecular and morphological designs of inorganic coatings have impact on the separator’s wettability and thus battery performance. Firmly attachment of ceramic coatings on the surface of separator with enhanced wettability is a huge challenge.

#### Inorganic Carbon-Based Nanomaterials

Inorganic carbon-based nanomaterials are widely used in the field of clean energy storage and conversion because of their unique crystal structure, rich chemical bond types and good environmental adaptability, including graphene [70, 73], carbon fiber (CFs) [76], carbon nanotubes (CNTs) [77, 85] and other graphitic carbons. On the one hand, carbon-based coatings can greatly enhance surface-wetting properties by changing the separator morphology and surface polarity without reducing the lithium-ion transport flux. On the other hand, their small pore sizes can decrease the local current density and lithium surface reaction to inhibit lithium dendrite formation. Meanwhile, carbon-based nanomaterials can synergistically improve the performance of other additives [70].

**Fig. 5:**
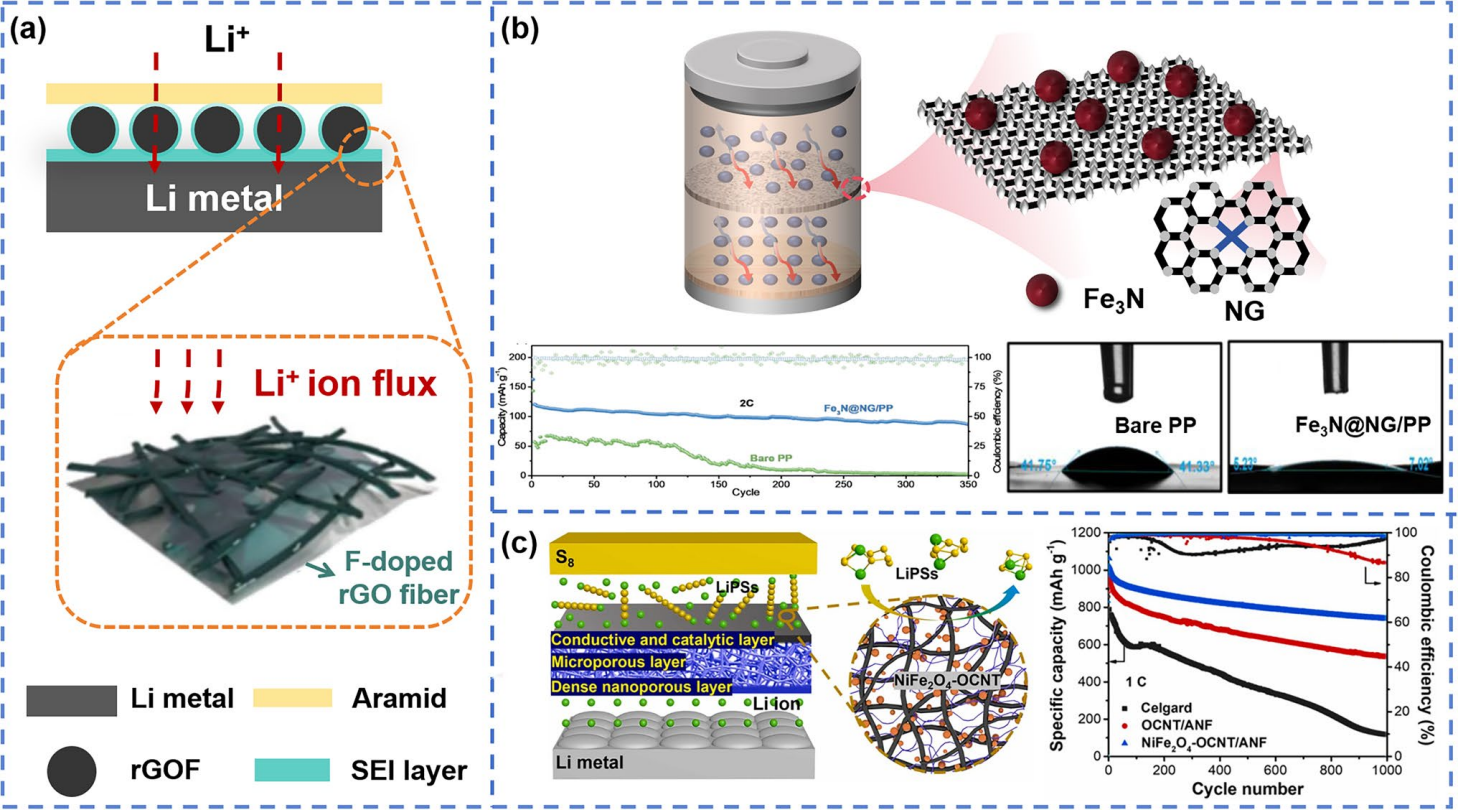
**a** Schematic illustration of ANF/rGOF separator [72]. Copyright 2020, WILEY-VCN. **b** Schematic illustration of the Fe_3_N@NG functionalized separator, contact angles and battery performance of bare PP and Fe_3_N@NG separators [75]. Copyright 2022, Elsevier. **c** Schematic illustration and battery performance of ANF/NiFe_2_O_4_-OCNT separator [77]. Copyright 2024, Elsevier

When the two-dimensional graphene oxide (GO) nanomaterial was exploited as a coating material, good electrolyte wettability (contact angle 15°) could be achieved for the coated separator [70]. The coated separator could be further functionalized for improved performance. Kown et al. [71] designed a GO-coated separator functionalized with polyacrylic acid (PAA), which showed better electrolyte wettability (the contact angle decreasing from 68° to 54.4°), improved ionic conductivity, and more homogeneous Li^+^ flux than the only GO-coated separator. When GO was doped with other inorganic materials and applied as coating materials, the obtained composite separator exhibited excellent wettability (contact angle no exceeding 10°) [73–75], high ionic conductivity (no less than 0.7 mS cm^–1^) [74, 75] and remarkable battery cycling performance. Yang et al. [73] creatively designed B/N co-doping reduced graphene oxide/boron nitride nanosheet, and exploited it for separator coating. This coated separator assembled lithium-sulfur batteries showed capacity retention of 92.3% after 500 cycles at a current density of 4C. The Laponite nanosheets and carbon black coated Celgard separator had good thermal stability at 160 °C, amazing electrolyte wettability (contact angle of nearly 0°), high ion conductivity (0.59 mS cm^–1^) and good flexibility [69]. In addition, the carbon material-modified separator showed promise in inhibition of lithium dendrite growth. The fabricated GO fibers were filtered on aramid paper, which were bonded together by hydrogen bonding. The rGO-coated aramid nanofiber (ANF) separator induced the formation of LiF, thus stabilizing the SEI layer, as shown in Fig. 5a [72]. The rGOF was able to capture of F^−^ produced by the decomposition of LiPF_6_ electrolyte, and promoted the formation of LiF rich SEI. The rGO/Li-Al-LDH composite nanosheets coating could not only improve the wettability of the LEs of the separator (contact angle of nearly 0°), but also provided additional ion channels, thus endowing more efficient lithium-ion transport characteristics for the separator [74]. The Fe_3_N-doped GO-coated separator inhibited the growth of lithium dendrites by accelerating Li^+^ transport and adjusting Li^+^ flux, as shown in Fig. 5b [75]. The CNT-modified ANF separator also has excellent wettability [77]. Meanwhile, its conductive and catalytic characteristics with NiFe_2_O_4_ modification can efficiently enhance the conversion of lithium polysulfides, as shown in Fig. 5c.

#### Other Inorganic Materials

Except for ceramic nanoparticles and inorganic carbon materials, some nitrides and sulfides are also exploited for separator modification due to their unique characteristics, such as high thermal stability and thermal conductivity. Thermal conductive separators can be fabricated by surface coating of hexagonal boron nitride (h-BN). In addition, the h-BN coated polypropylene (PP) separator induced the formation of SEI with more inorganic species and improved the cycling performance of battery [79].

## Summary

Introducing inorganic materials on the surface of separators effectively improves the electrolyte wettability and ionic conductivity. However, inorganic materials still show some shortcomings. Firstly, the coating thickness of separator can hardly be tuned and can easily reach several micrometers, which greatly increases the weight of separators and decreases the specific energy density of batteries. Secondly, the polymer binders tend to block the pores of separators, which sacrifice the ionic conductivity and power capability of batteries. Lastly, the polarity difference and the weak adhesion between the matrix separator and the coating layers make the shedding problem difficult to ignore, which affects the battery cycling life.

### Organic Framework Coated Separator

Metal–organic frameworks (MOFs) and covalent-organic frameworks (COFs) have attracted tremendous attention in various areas due to their structural adjustability, extensive specific surface area, and well-organized pores [86]. Compared with the above inorganic nanomaterials, the porous structures of organic frameworks are more regular and uniform, and their pore size is more controllable [87]. When MOFs and COFs are coated on separators, their abundant and ordered nanopores and relatively large specific surface areas increases the contact area with electrolyte. Meanwhile, the nanopores can retain the electrolyte for a longer time, resulting in good electrolyte wettability and retention capability. In addition, this structural characteristic enables selective transport and uniform deposition of Li^+^ while improves the wettability and ionic conductivity of the separators [87–91]. The characteristics and electrochemical performances of some representative organic framework-coated separators developed in recent years are summarized in Table 3.

**Table 3:** Partial summary of characteristics and electrochemical performance of organic framework coated separators

Separator	Coating thickness (μm)	Electrolyte contact angle	Electrolyte uptake	Ionic conductivity (mS cm^–1^) at RT	Li^+^ transference number	Cyclic performance	References
PP/MOFs	–^a^	15.88°	–	0.26	0.68	98.1% after 50 cycles at 2C (modified)	[92]
PP/UIO-SOLi	4	0°	–	0.11	0.82	66.7% after 170 cycles at 0.1C (original) 97.0% after 640 cycles at 0.1C (modified)	[93]
PP/Cr-MOFs	22	9.52°	250%	3.50	–	–	[94]
PE/COF-C16	1.4	16.02°	–	–	–	90.0% after 800 cycles at 1C	[95]
PP/COF-COOH	10	13.98°	–	0.64	0.70	–	[96]
PP/COF-F	< 10	12°	–	0.76	0.87	80.1% after 2000 cycles at 5C	[97]
PP/COF	5	26.9°	224%	0.24	0.65	88.5% after 200 cycles at 1C (original) 97.8% after 200 cycles at 1C (modified)	[98]
PP/PA-COF	8.2	–	–	–	0.87	93.6% after 200 cycles at 0.5C	[99]
PE/HC-CTF	1.5	41.2°	124%	0.67	0.60	16.8% after 300 cycles at 1C (original) 45.4% after 1000 cycles at 1C (modified)	[100]

^a^ “–”means not mentioned

**Fig. 6:**
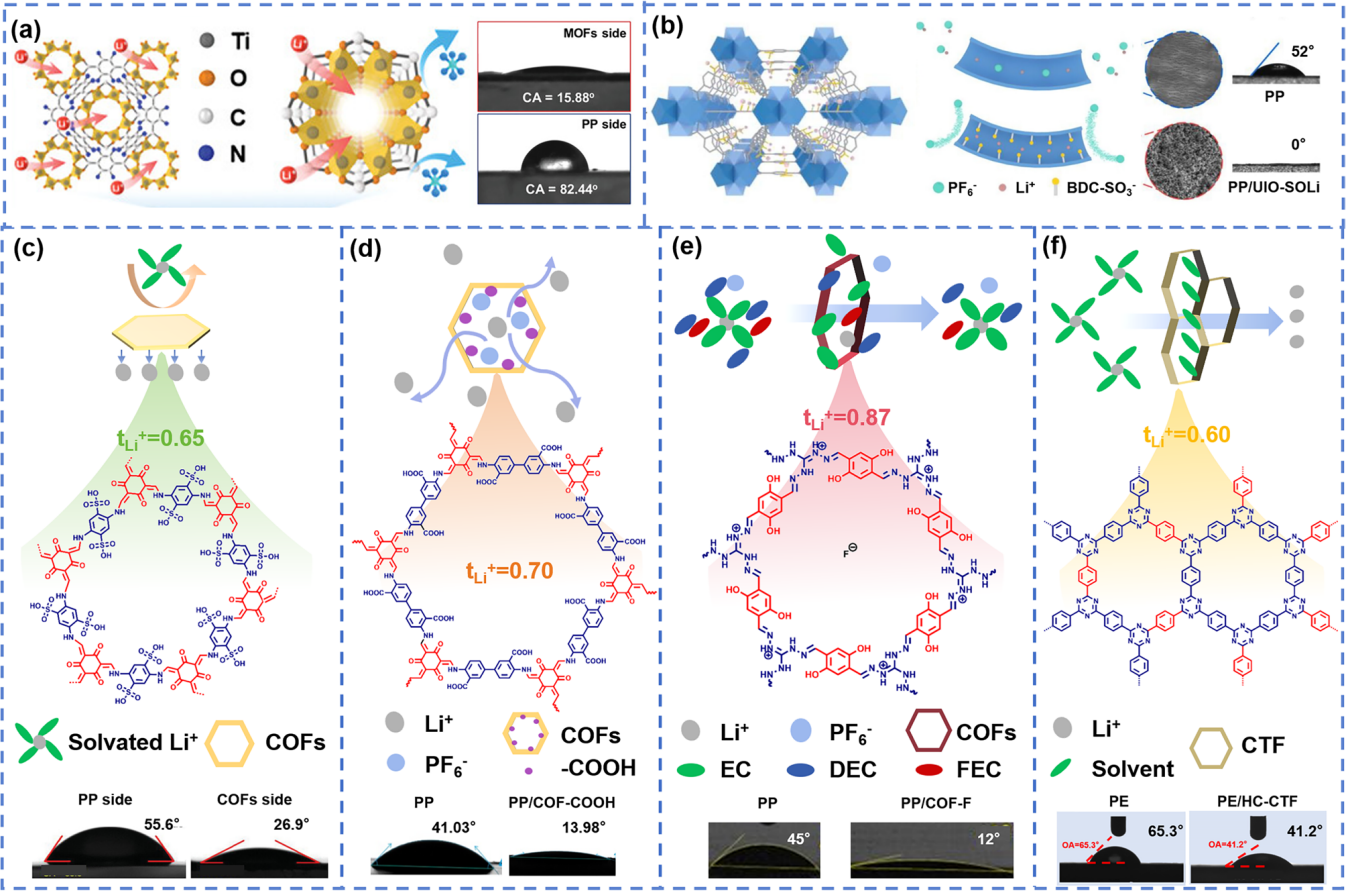
**a**, **b** Schematic illustration of PP/MOF separators mechanism and static electrolyte contact angle [92, 93]. Copyright 2021, WILEY-VCN. Copyright 2023, WILEY-VCN. **c**–**f** Schematic illustration of PP/COF separators mechanism, static electrolyte contact angle, and Li^+^ transference number [96–98, 100]. Copyright 2022, WILEY-VCN. Copyright 2023, WILEY-VCN. Copyright 2023, Elsevier. Copyright 2024, OAE

A large number of C, O, and N atoms in the framework structure provides active sites for further modification [89], and some polar negatively charged groups such as − SO_3_H, − COOH, and − F ions have been successfully attached to the framework structure. Commonly, these frameworks have been used for surface coating of the commercial polyolefin separator. Hao et al. [92] carefully designed MOFs containing both − NH_2_ and − SO_3_^−^ groups. It showed good cation-selectivity and excellent electrolyte wettability with contact angle of 15.88°, as shown in Fig. 6a. The intrinsic nanochannels and negatively charged intergranular channels in the MOF structure limited the free migration of anions, and the rapid and uniform deposition of Li^+^ inhibited the lithium dendrites growth. The capacity retention ratio reached 98.08% after 50 cycles at 2C. The composite separator coated with TpPa-2SO_3_H COFs had both neatly arranged nanochannels and rich functional groups. It possessed a suitable porous structure, high electrolyte absorption and improved electrolyte wettability, providing rich pathways for rapid Li^+^ transport, as shown in Fig. 6b, c [93, 98]. An et al. [94] designed a covalent organic framework modified with lithium-philic carbonyl and carboxy groups (COF-COOH) and coated it on PP (Fig. 6d). The COF-COOH presented a crystalline two-dimensional structure with abundant pores and a large number of lipophilic groups, which was conducive to improving the electrolyte wettability (contact angle 13.98°) and endowing high ionic conductivity (0.64 mS cm^–1^) of the separator. At the same time, the presence of negative charge sites in COF-COOH inhibited the diffusion of anions in lithium salts through electrostatic interaction, and Li^+^ transference number increased to 0.70. Yao et al. [98] used a cationic COF and weakly bonded fluoride ion (F^−^) for separator modification, which not only improved the electrolyte’s wettability but also constructed a robust LiF-rich SEI, as shown in Fig. 6e. Interestingly, the highly ordered crystalline covalent triazine frameworks (CTF) also contributed to selective and fast transport of Li^+^, as shown in Fig. 6f [100].

### Inorganic–Organic Coated Separators

Introducing hydrophilic polymers on the surface of separators can enhance their surface energy. Hydrophilic functional groups in polymers improve electrolyte wettability to accelerate Li-ion conduction. Moreover, polymer coatings can introduce highly electronegative functional groups, such as carboxyl and sulfonates, and endue a net negative surface charge on the surface of separators. It can not only retard the migration of anions by electrostatic repulsive but also enable the rapid transport of Li^+^, thus efficiently suppressing the growth of lithium dendrites [101]. Poly-*p*-phenylene terephthamide (PPTA) could be coated on the commercial PP separator without binders. The electrolyte wettability was greatly improved and the electrolyte contact angle was reduced from 50° to 15° after coating [102]. Polymers such as polytetrafluoroethylene (PVDF) [103, 104] and poly(ethylene oxide) (PEO) [105] have also been used to modify separators for improved wettability. However, the thickness of the polymer coating is difficult to control, and thick coating easily causes blockage of the separator pores and reduction of ionic conductivity. The ionic conductivity of polymers such as polydopamine [106] is also low, and the mechanical properties are poor, which affects the battery cycle performance. The characteristics and electrochemical performance of some representative polymer-coated separators are summarized in Table 4.

**Table 4:** Summary of characteristics and electrochemical performance of polymer-coated separators

Separator	Coating thickness (μm)	Porosity^a^	Electrolyte contact angle	Electrolyte uptake	Ionic conductivity (mS cm^–1^) at RT	Cyclic performance	References
PE/PVDF-HFP	–^b^	–	–	140%	0.44	–	[103]
PE/PMMA	2 ~ 3	–	–	200%	0.52	–	[107]
PE/PDA	–	–	39°	126%	0.41	–	[27]
PE/EC	2 ~ 3	–	–	–	0.68	–	[108]
PE/AN-*co*-MMA	–	–	–	150%	2.06	83.0% after 50 cycles at 0.2C (original) 86.3% after 50 cycles at 0.2C (modified)	[109]
PP/PDA	–	–	< 20° (water)	290%	1.30	94.2% after 100 cycles at 0.2C	[110]
Cellulose/PVDF-HFP	–	65%	55°	280%	1.04	71.0% after 100 cycles at 0.5C (PP) 83.0% after 100 cycles at 0.5C (modified)	[111]
PE/PDA	–	–	39°	112%	0.30	94.9% after 100 cycles at 0.5C	[40]
PE/PEO	–	–	–	76%	1.00	–	[105]
PP/PI	8.16	53.05%	5°	208%	0.34	75.4% after 200 cycles at 1C (original) 80.1% after 200 cycles at 1C (modified)	[112]
PE/PBI	4	54%	10°	225%	0.60	93.9% after 100 cycles at 0.5C (original) 97.7% after 100 cycles at 0.5C (modified)	[113]
PE/PI	–	60%	0°	400%	1.34	96.4% after 100 cycles at 1C	[114]
PVDF/PMIA	–	83.97%	–	–	0.81	84.3% after 100 cycles at 0.2C 93.1% after 100 cycles at 0.2C	[115]
PEEK/PMMA	–	64.1%	0°	173%	1.03	–	[116]

^a^ “Porosity” is the entire separator’s, instead of the coating’s. ^b^ “–” means not mentioned

To solve the shortcomings of inorganic material-coated and polymer-coated separators, researchers devoted much efforts to developing inorganic–organic composite-coated separators. The composite coatings can effectively improve the overall performance of the separators in high-energy storage batteries. The characteristics and electrochemical performance of inorganic–organic composite coated separators are summarized in Table 5.

**Table 5:** Summary of characteristics and electrochemical performance of inorganic–organic coated separators

Separator	Coating thickness (μm)	Porosity^a^	Electrolyte contact angle	Electrolyte uptake	Ionic conductivity (mS cm^–1^) at RT	Cyclic performance	References
PE/Al_2_O_3_/DLSS	6	–^b^	63.11°	100%	0.85	89.2% after 400 cycles at 0.5C 93.6% after 400 cycles at 0.5C	[117]
PP/GO-*g*-PAM	–	–	0°	430%	–	–	[118]
PE/Al_2_O_3_/PHC	1.5	42.7%	–	92%	0.93	83.4% after 350 cycles at 1C (original) 87.6% after 350 cycles at 1C (modified)	[119]
PP/SiO_2_-PVA	–	50.5%	12.7°	201%	1.26	73.6% after 100 cycles at 0.5C (original) 91.0% after 100 cycles at 0.5C (modified)	[120]
PE/Al_2_O_3_/PDA	4	35.3%	0°	70%	0.71	–	[121]
PE/PVDF-EC-A-SiO_2_	2	–	23.2°	–	0.79	91.7% after 200 cycles at 0.5C (original) 95.7% after 200 cycles at 0.5C (modified)	[122]
PE/SiO_2_-PMMA	5	–	6.1°	90%	1.08	94.6% after 100 cycles at 1C (original) 95.4% after 100 cycles at 1C (modified)	[123]
PE/ZrO_2_@PI	4	54.28%	0°	164%	0.68	75.1% after 200 cycles at 1C (original) 84.4% after 200 cycles at 1C (modified)	[124]
PE/SiO_2_-PZS	1.5	–	13.4°	155%	1.04	80.0% after 100 cycles at 2C (original) 81.7% after 100 cycles at 2C (modified)	[125]
PP/PDA@AlN	18	–	0°	–	0.75	–	[106]
PP/PDA/Gr-CMC	21	–	–	–	–	58.0% after 1000 cycles at 1C (original) 93.3% after 1000 cycles at 1C (modified)	[126]
PVDF/PAN/Al_2_O_3_/NC	5	–	0° (H_2_O)	395%	1.49	57.1% after 120 cycles at 0.5C (original) 65.9% after 120 cycles at 0.5C (modified)	[127]
PPS/SiO_2_/PVDF-HFP	–	–	–	230%	1.02	–	[128]
PP/PI/Al_2_O_3_	2.5	–	–	–	0.95	100% after 350 cycles at 0.5C	[129]

^a^ “Porosity” is the entire separator’s, instead of the coating’s. ^b^ “–” means not mentioned

**Fig. 7:**
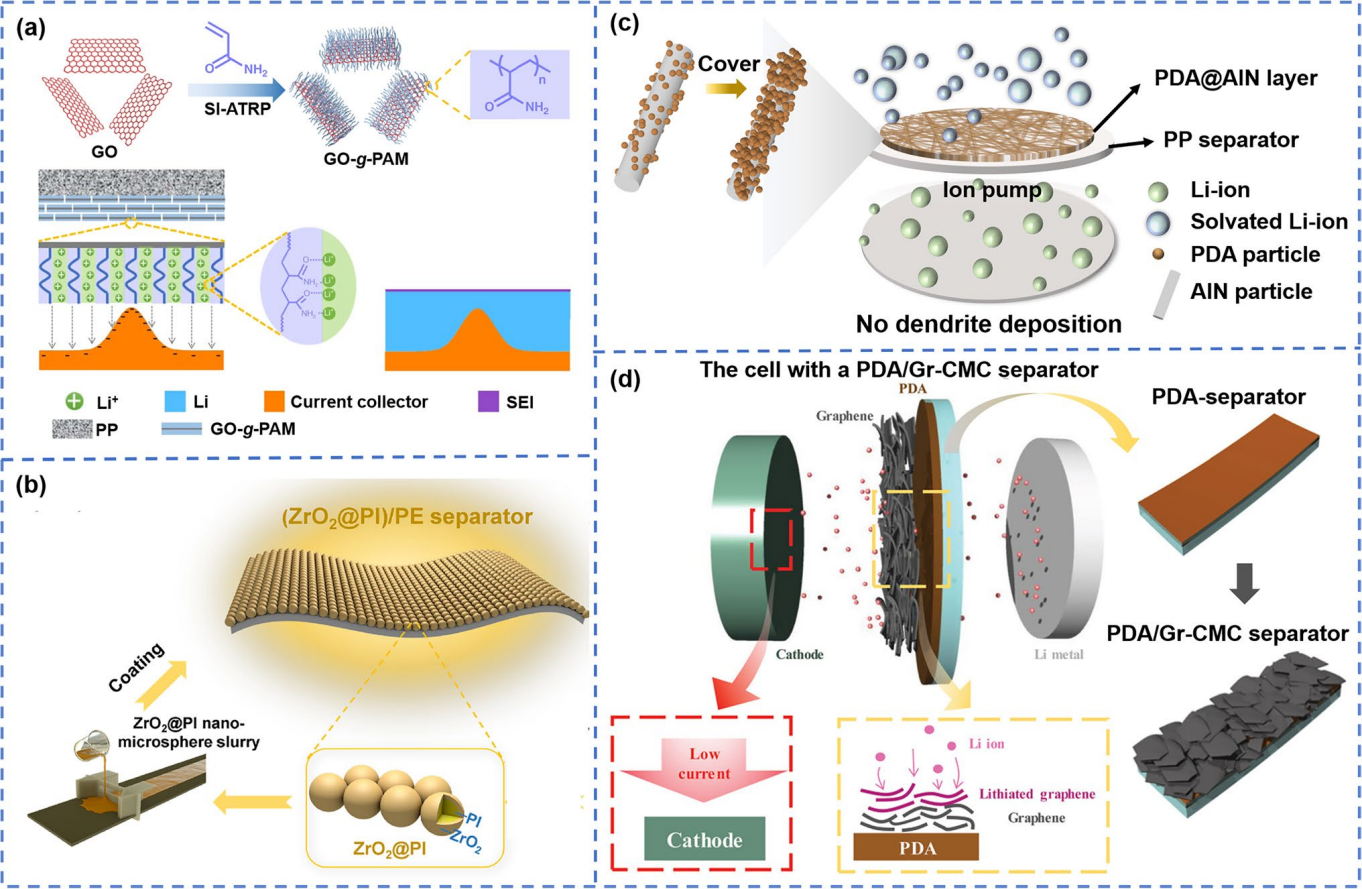
**a** Schematic illustration of the synthesis of GO-g-PAM molecular brushes and lithium deposition on anode using GO-g-PAM/PP separator [120]. Copyright 2019, Springer Nature. **b** Schematic illustration of the preparation of the ZrO_2_@PI/PE separator [124]. Copyright 2022, Elsevier. **c** Schematic illustration of the PDA@AlN/PP separator [106]. Copyright 2024, WILEY-VCN. **d** Schematic illustration of the role of PP/PDA/Gr-CMC separator on the electrochemical reactions during discharging [126]. Copyright 2018, WILEY-VCN

Coating slurries with both inorganic and organic materials on the surface of the separator is a commonly used method [127]. For example, when polymeric surfactant disodium laureth sulfosuccinate (DLSS) was introduced into the Al_2_O_3_ slurry, the polymer with both hydrophilic and hydrophobic groups not only enabled the coating with water-based slurry, but also greatly improved the electrolyte wettability (contact angle decreasing from 199.27° to 63.11°) and electrolyte uptake (100%) [117]. Li et al. [118] grafted GO onto polyacrylamide (PAM), while Zhang et al. [120] fabricated covalently bonded ceramic nanoparticles-poly(vinyl alcohol) (PVA) composites, and then coated it on the separator. On the one hand, the polar groups on the surface of the separator were increased, on the other hand, the coating materials had tight adhesion and uniformity of the coating materials was achieved. Combined with high electrolyte wettability and ionic conductivity, the cycling performance of the battery was largely improved, as shown in Fig. 7a. Wang et al. [124] prepared ZrO_2_@PI hollow core–shell microspheres and coated it on the polyolefin separator, as shown in Fig. 7b. The separator has excellent wettability and ionic conductivity (0.68 mS cm^–1^), and the hollow microspheres reduced the surface weight density of the separator, resulting in a high overall energy density of the battery. Fu et al. [125] coated core–shell structured SiO_2_-PZS (silica-poly (cyclotriphosphazene-*co*-4,4′-sulfonyldiphenol)) nanoparticles on the separator, and the hydroxyl groups and N, O atoms on the surface could coordinate with Li^+^ to enhance the dissociation of lithium salt (LiPF_6_), thus further enhancing the ionic conductivity (1.04 mS cm^–1^) and showing higher wettability. Different from firstly fabrication of inorganic and organic composites and then coating on matrix separator, Tang et al. [106] coated the inorganic material AlN on the separator at first, and then grew polydopamine (PDA) in situ on the coating, as shown in Fig. 7c. The combination of rigidity and flexibility of the separator was realized by optimization of the modification sequence. The integration of the two materials with different characteristics provided another strategy to enable fast ion transport and enhanced wettability, benefiting for uniform lithium deposition. Kim et al. [126] first deposited organic polydopamine (PDA) on the PP separator, and then modified the inorganic graphene (Gr) nanosheets. The prepared three-layer separator had excellent electrolyte wettability, enhanced coulombic efficiency and increased capacity of lithium storage with 93.3% capacity retention after 1000 cycles at 1C. With this separator, the interfacial impedance between the electrode and the separator was significantly reduced, as shown in Fig. 7d.

### Section Summary

A variety of inorganic and organic materials can be easily modified on the separator by surface coating. The adhesion of inorganic materials on the commonly used polyolefin is weak, and the binder tends to melt resulting in the blockage of the pores. In some cases, the inorganic materials can be coated without binder by preprocessing of the matrix separator, such as ultraviolet electron beam irradiation. The thickness of the polymer coating is not controllable, and the falling-off problem still exists. It is expected that in situ modification or grafting should be better strategies for polymer coating. The coating of inorganic–organic composite can combine the functions of both materials to a certain extent, and has obvious improvement in wettability and ion conductivity of the separators. In addition, the adhesion of coating layer with the matrix separator and the dimensional stability of the coated separator also increased, leading to highly improved cycling stability of the assembled batteries.

## In Situ Modified Separator

While surface coating is indeed a straightforward approach that enhances wettability with electrolytes, thereby improving electrochemical performance, its effectiveness is constrained by the limited adhesion between the coating and the base separator, as noted in previous studies [130–134]. In contrast, in situ modification emerges as a superior alternative. This strategy not only achieves a comparable level of surface wettability akin to surface coating but also ensures a robust bond between the coating materials and the base separator. Furthermore, surface coating typically covers only the outer surface of the base separator. Conversely, coatings formed through in situ modification can permeate the entire surface, both external and internal, of the separator [135, 136]. Crucially, the uniformity of the in situ-grown coating significantly minimizes interfacial resistance and facilitates the homogeneous distribution of Li^+^. Additionally, in situ modification has the advantage of producing thin coatings while preserving the pore structure of the base separator, as evidenced in various studies [137–140].

### In Situ Formed Inorganic Modification Materials

Inorganic materials such as SiO_2_ [56], ZrO_2_ [141] and Al_2_O_3_ [142], among others, are widely favored as modification materials owing to their notable enhancements in wettability and thermal stability. Nevertheless, their weak interaction with the base separator necessitates the use of organic binders to bolster adhesion. Furthermore, the incorporation of ceramic particles can result in an unwanted increase in both the thickness and weight of the separator [112, 143–145]. In situ modification can solve these problems because the inorganic coating via in situ growth distributes uniformly and adheres tightly to the base separator while not significantly affecting the pore structure and separator thickness [146, 147]. The characteristics and electrochemical performance of in situ modified separators by inorganic materials are listed in Table 6.

**Table 6:** The characteristics and electrochemical performance of in situ modified separators by inorganic materials

Separator	Coating thickness	Porosity^a^ (%)	Coating mass fraction (%)	Electrolyte contact angle	Electrolyte uptake	Ionic conductivity (mS cm^–1^) at RT	Cyclic performance	References
PP/Al_2_O_3_ (ALD)	6 nm	44	40.1	–^b^	–	–	80% after 1000 cycles at 4C	[148]
PVDF/Al_2_O_3_ (ALD)	10 nm	87	-	22°	350%	3.1	91% after 100 cycles at 0.2C (original) 100% after 100 cycles at 0.2C (modified)	[149]
PE/PP/PE/Al_2_O_3_ (ALD)	5 nm	49	8.5	34.5°	260%		75% after 100 cycles at 1C (original) 85% after 100 cycles at 1C (modified)	[150]
PP/TiO_2_ (ALD)	1 nm	41	3.5	38.4°	–	–	91% after 100 cycles at 5C	[151]
PI/SiO_2_	–	61	14.3	6.8°	264%		88% after 100 cycles at 1C	[152]
PI/ZrO_2_	–	83	7.65	18°	345%	3.73	100% after 100 cycles at 1C	[153]
CNFs/Sn-MoS_2_	–	–	-	–	–	–	51% after 500 cycles at 2C (original) 73% after 500 cycles at 2C (modified)	[154]
PP/CoSO_2_	12.1 μm	–	-	–	–	–	46% after 500 cycles at 1C (original) 62% after 500 cycles at 1C (modified)	[155]
PP/GDY	< 2 μm	–	-	–	–	–	< 10% after 500 cycles at 1C (original) 53% after 500 cycles at 1C (modified)	[156]

^a^ “Porosity” is the entire separator’s, instead of the coating’s. ^b^ “–” means not mentioned

**Fig. 8:**
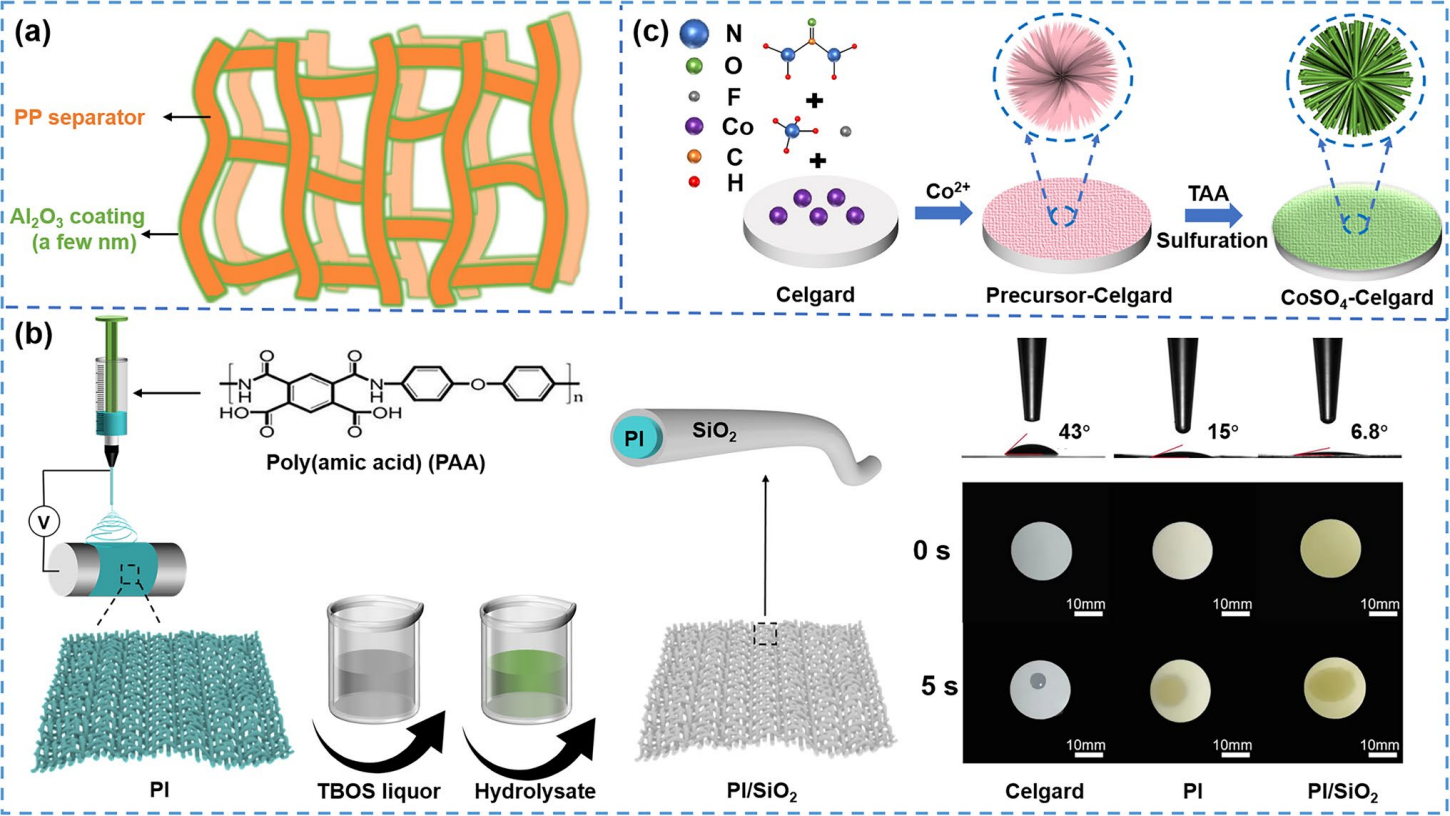
**a** Thin Al_2_O_3_ ALD coating (a few nm) on the PP separator [148]. Copyright 2012, WILEY–VCH. **b** Surface SiO_2_ nano-encapsulation of PI nanofiber separator by in situ nano-encapsulation hydrolysis method [152]. Copyright 2021, Elsevier. **c** Schematic illustration of the fabrication procedure of hydrated CoSO_4_-Celgard arrays [155]. Copyright 2020, Elsevier

Atomic layer deposition (ALD) technology is a method for depositing layers on the substrate surface in the form of single atomic films. This method allows for the creation of ultra-thin and conformal coating layers, which exhibit precise thickness control and exceptional uniformity. By utilizing ALD, inorganic materials can grow conformally within the internal microstructure of the substrate. [157–160]. For instance, Jung et al. [148] deposited the atomic layers of Al_2_O_3_ on the surface of PP separator by utilizing trimethylaluminium (TMA) and H_2_O as precursors. As shown in Fig. 8a, the pore structure was almost unchanged because of the slow growth rate of Al_2_O_3_ on PP (increasing 1.2 Å per cycle). The Al_2_O_3_ ALD coating significantly enhanced the wettability and the LiPF_6_ in pure propylene carbonate (PC) could completely infiltrate the modified separator (after 50 and 100 ALD cycles). Therefore, the nano-Li_4_Ti_5_O_12_ (LTO, anode)/LiFePO_4_ (LFP, cathode) half-cell with after 50 ALD cycles separator showed excellent cycling stability (a high-capacity retention of 80% at 4C after 1000 charge–discharge cycles). The ALD technology can precisely control the thickness and uniformity of coating layers at the angstrom level, but the required test device is expensive. Besides, a pristine PP separator surface usually requires large numbers of ALD cycles [161, 162]. Therefore, its large-scale application is limited.

In situ hydrolysis of inorganic material precursor on the nonwoven separator is also commonly used, forming a unique core–shell structure to improve the wettability and mechanical properties of nanofibers. For instance, Wu et al. [152] proposed a novel idea of in situ nano-encapsulation hydrolysis method to prepare uniform silica nanolayers on polyimide (PI) nanofibers (Fig. 8b). First, they prepared PI nanofibers through electrospinning, and then immersed it in the silica precursor solution. After heating and hydrolysis, the silicon grew uniformly and densely in situ on the surface of the PI nanofiber and formed a core–shell structure. This method can effectively encapsulate inorganic materials on the inner and outer surfaces of the separator without a significant increase in thickness. Due to the coverage of hydrophilic silica on the surface, the wettability of the separator (contact angle of only 6.8°) and the rate of electrolyte absorption (absorbing liquid electrolyte completely in 5 s) are significantly improved. The in-situ generated silica effectively improves the wettability of the separator, which has a positive impact on improving the transmission capacity and efficiency of Li^+^. The NCM811/Li half-cell with modified separator showed a high capacity of 146 mAh g^−1^ at 5C, and a 10% increase was achieved when compared with the PI separator. Furthermore, the battery with PI/SiO_2_ nanofiber separator maintained a high-capacity retention rate of 88% after 100 cycles at 1C. Similarly, Dong et al. [153] reported a zirconium dioxide (ZrO_2_)-armored polymeric separator by in situ hydrolysis of ZrOCl_2_ on PI nanofibers, forming a core–shell structure. The introduction of ZrO_2_ shell layers promoted the wettability (contact angle of 18°) of the separator and rate capability (128.6 mAh g^−1^ at 5C) of the assembled batteries.

Some special inorganic materials can be grown in situ on separators through methods such as the hydrothermal reaction. For instance, Lu et al. [155] prepared CoSO_4_ hydrate nano-architecture in situ on the surface of the PP separator through the hydrothermal reaction (Fig. 8c). The polar CoSO_4_ can catalyze the conversion of polysulfide during charging and discharging, thus improving the utilization of sulfur. Besides, Rich adsorption sites ensure sufficient Li^+^ transport while suppressing shuttle effects. Therefore, the cell with the modified separator showed a high initial specific capacity of 807.7 mAh g^−1^ and maintained a capacity retention rate of 62.5% after 500 cycles at 1C.

Generally speaking, inorganic materials can significantly enhance the wettability, thermal stability, and mechanical properties of separators. However, there is a scarcity of reports on the in situ modification of separators using inorganic materials, potentially due to the intricate nature of their in situ preparation process.

### In Situ Formed Organic Framework Materials

When compared to other methods [163–167], the in situ formation of MOF and COF materials on the separator offers several advantages: it enhances adhesion to mitigate stripping defects, reduces interfacial resistance, and enables the attainment of thinner, more uniform coatings, thereby augmenting energy density. The characteristics and electrochemical performance of in situ modified separators by MOF as well as COF are listed in Table 7.

**Table 7:** The characteristics and electrochemical performance of in situ modified separators by MOF as well as COF

Separator	Coating thickness	Porosity^a^ (%)	Electrolyte contact angle	Electrolyte uptake	Ionic conductivity (mS cm^–1^) at RT	Cyclic performance	References
PEN/PDA + ZIF-67	–^b^	–	0°	450%	1.57	83% after 240 cycles at 0.5C (original) 96% after 240 cycles at 0.5C (modified)	[168]
GO-PAN/MOF	< 10 nm	–	0°	–	–	63% after 100 cycles at 1C (original) 81% after 600 cycles at 5C (modified)	[169]
PP/Ni_3_(HITP)_2_	340 nm	–	–	–	-	38% after 100 cycles at 0.2C (original) 84.1% after 500 cycles at 1C (modified)	[170]
PVDF-PMIA/ZIF-8	–	–	15.8°	1908%	1.66	75% after 300 cycles at 0.2C (original) 73% after 300 cycles at 0.2C (modified)	[171]
PP/COF-SO_3_H	127 nm		16.4°	–	0.18	67% after 500 cycles at 1C (original) 75% after 500 cycles at 1C (modified)	[136]
CNT/COF-2S	–	49	0°	–	–	53% after 500 cycles at 1C (original) 84% after 500 cycles at 1C (modified)	[172]
PP/PDA + SWCNT + COF	669 nm	–	14.8°	–	0.53	43% after 500 cycles at 1C (original) 74% after 500 cycles at 1C (modified)	[173]
PAN/CTP + LLZTO	100 nm	65.8	8.5°	385%	0.64	33% after 500 cycles at 0.5C (original) 70.3% after 500 cycles at 0.5C (modified)	[140]

^a^ “Porosity” is the entire separator’s, instead of the coating’s. ^b^ “–” means not mentioned

**Fig. 9:**
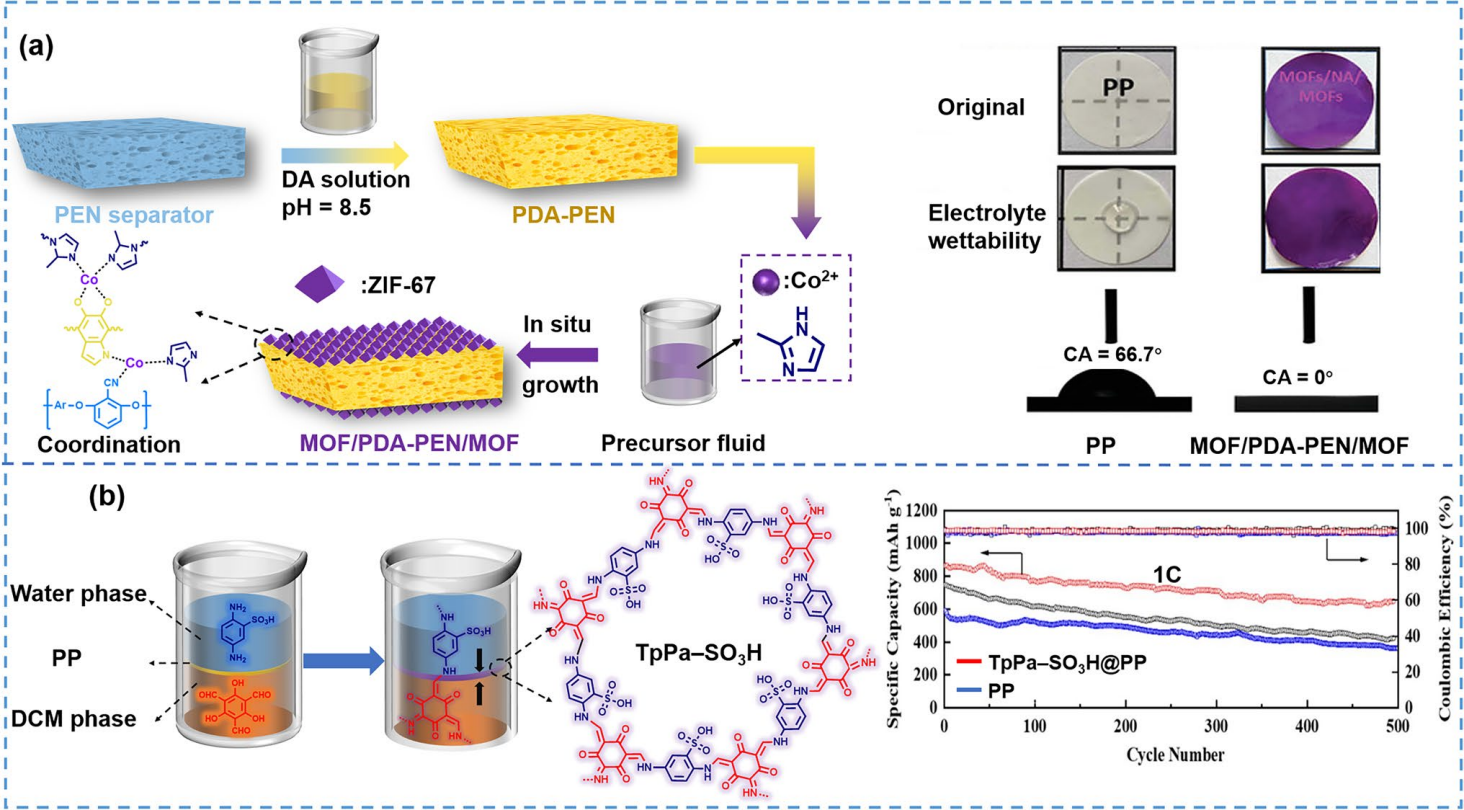
**a** Schematic for construction of sandwich-structured MOF/PDA-PEN/MOF hybrid separators [168]. Copyright 2022, WILEY–VCH. **b** Schematic of the synthesis of COF modified separator by an in situ interface method [136]. Copyright 2022, Elsevier

MOF was considered to be more effective candidates for regulating Li^+^ distribution and inhibiting lithium dendrites [174–177]. For instance, the Zeolitic imidazolate framework (ZIF), as a MOF subclass, features zeolite topologies generated from metal ions and imidazolate ligands. Because of its high porosity, good thermal and chemical stabilities, the feasibility of in situ growth on polymer substrates as well as anion trapping capacity, ZIF had been selected as a representative MOF to modify the separator [178–180]. Lin et al. [168] prepared a functional separator, which ZIF-67 grew in situ on both sides of the dopamine (DA) pretreated polyarylene ether nitrile (PEN) separator (PEN@PDA) shown in Fig. 9a. Specifically, based on the self-polymerization of DA and the π-π interaction between PDA and PEN, PDA tightly adhered to the surface of PEN while introducing abundant reactive groups to couple with Co^2+^ and 2-methylimidazole ligands. As a result of the stronger negative charge of the PEN@PDA separator, it effectively adsorbed Co^2+^ and formed active sites for ZIF-67 layer growth, which facilitated the stable fixation of a homogeneous and defect-free coating on the outer and inner surface of the separator. As a highly porous material, the in situ growth of ZIF-67 increased significantly the porosity and overall specific surface area of the separator. Therefore, the contact angle between the electrolyte and modified separator was almost 0°. The electrolyte absorption rate of the separator reached 450%. After being treated at 50 °C for 500 min, 70 wt% of the electrolyte within the separator can be maintained. The above abilities of separator enabled its greatly improved ionic conductivity compared with the base separator (from 0.86 to 1.57 mS cm^−1^). Besides, ZIF-67 endowed the separator with a uniform sub-nano/nanoporous structure and ability to adsorb anions, which homogenized the concentration of electrolyte at the electrode interface and guided uniform Li^+^ diffusion. It ultimately resulted in a stable SEI with high ionic conductivity. Hence, the cell with the modified PEN separator showed a high *t*_Li_^+^ of 0.81, superior rate capability, and cycle stability (specific capacity of 152 mAh g^−1^ and capacity retention of 96.3% after 240 cycles at 0.5C).

COF is a framework material with regular pores or holes connected by organic units with covalent bonds [88, 181, 182]. Zhao et al. [136] prepared a COF modified PP separator by in situ interfacial polymerization, which not only avoided significant increases in thickness and volume of the separator but also reduced the interfacial resistance (Fig. 9b). The PP separator was placed at the interface of the dichloromethane (DCM) phase and the water phase, then 1,3,5-triformylphloroglucinol (Tp) molecules in the DCM phase and 2,5-diaminobenzene sulfonic acid (Pa-SO_3_H) molecules in the water phase met at the PP separator with the diffusion of liquid flow. The COF consisted of Tp and Pa-SO_3_H units grew in situ on both the inner and outer surfaces of the separator. The introduction of lipophilic groups-sulfonic groups inhibits the shuttle of soluble polysulfides by Coulomb repulsion while promoting lithium-ion migration. Besides, sulfonic groups enhanced the wettability with electrolyte (contact angle decreasing from 42.7° to 16.4°), which resulted in a lower interfacial resistance and higher ionic conductivity. With sulfonic groups and regular channels of TpPa-SO_3_H COF, the modified separator showed better electrochemical performance and battery performance than that of PP separator. The initial specific capacity of the cell with modified separator was 863.97 mAh g^−1^ at 1C, increasing by 56% compared with the PP separator (551.84 mAh g^−1^). Moreover, it was able to steadily work for 500 cycles with a high-capacity retention of 75%.

In summary, MOF and COF are excellent modification materials. Their regular channels regulate Li^+^ transport, and ultra-high specific surface areas improve wettability as well as electrolyte absorption rate. The flexible structural design expands the application range. Besides, in situ growth of MOFs and COFs overcomes the problem of poor adhesion.

### In Situ Formed Organic Modification Materials

To address the issue of poor wettability in commercial polyolefin separators, numerous hydrophilic polymers, including PEO [183], polymethyl methacrylate (PMMA) [184], PVDF [185], and PI [186], have been investigated. Most of these polymers are applied to the separator surface through solution-based coating processes. However, these methods often result in a reduction in porosity due to pore blockage and limited control over coating thickness, ultimately leading to a decrease in energy density [45, 107]. In situ modification emerges as a promising alternative strategy, as it preserves the porous structure while enhancing the adhesion between the coating and the base separator. The characteristics and electrochemical performance of in situ modified separators by organic modification materials are in Table 8.

**Table 8:** The characteristics and electrochemical performance of in situ modified separators by organic modification materials

Separator	Coating thickness	Porosity^a^ (%)	Coating mass fraction (%)	Contact angle	Electrolyte uptake	Ionic conductivity (mS cm^–1^) at RT	Cyclic performance	References
PE/PDA	–^b^	–	–	39° (H_2_O)	126%	0.41	–	[27]
PVDF/PDA	< 1 μm	72.8	11.7	0° (electrolyte)	254%	1.40	98.8% after 100 cycles at 1C (original) 99.1% after 100 cycles at 1C (modified)	[187]
CP/PDA	–	–	–	0° (electrolyte)	320%	1.29	< 50% after 600 cycles at 5C (original) > 80% after 600 cycles at 5C (modified)	[188]
PP/TA	–	–	–	72° (H_2_O)	125%	0.46	91% after 200 cycles at 1C	[189]
PP/PA	800 nm	41	1.8	54.7° (H_2_O)	129%	0.57	91.6% after 200 cycles at 1C (original) 97.7% after 200 cycles at 1C (modified)	[190]
PP/PEI + PDA	–	–	–	46.3° (H_2_O)	144%	0.58	65% after 200 cycles at 0.2C (original) 80% after 200 cycles at 0.2C (modified)	[191]
PP/PEI + TA	–	61.6	–	12.7° (electrolyte)	140%	0.95	69.3% after 200 cycles at 1C (original) 90.9% after 200 cycles at 1C (modified)	[192]
PP/PDA + PPFPA	–	–	–	36.5° (electrolyte)	209%	0.83	52% after 700 cycles at 1C (original) 83% after 700 cycles at 1C (modified)	[193]
PP/NCMP	–	–	–	< 5° (electrolyte)	116%	1.10	40% after 400 cycles at 0.2C (original) 76% after 800 cycles at 0.2C (modified)	[135]
PP/Al-CPP	800 nm	–	–	22.6° (electrolyte)	124%	0.51	40% after 500 cycles at 1C (original) 61% after 500 cycles at 1C (modified)	[194]

^a^ “Porosity” is the entire separator’s, instead of the coating’s. ^b^ “–”means not mentioned

**Fig. 10:**
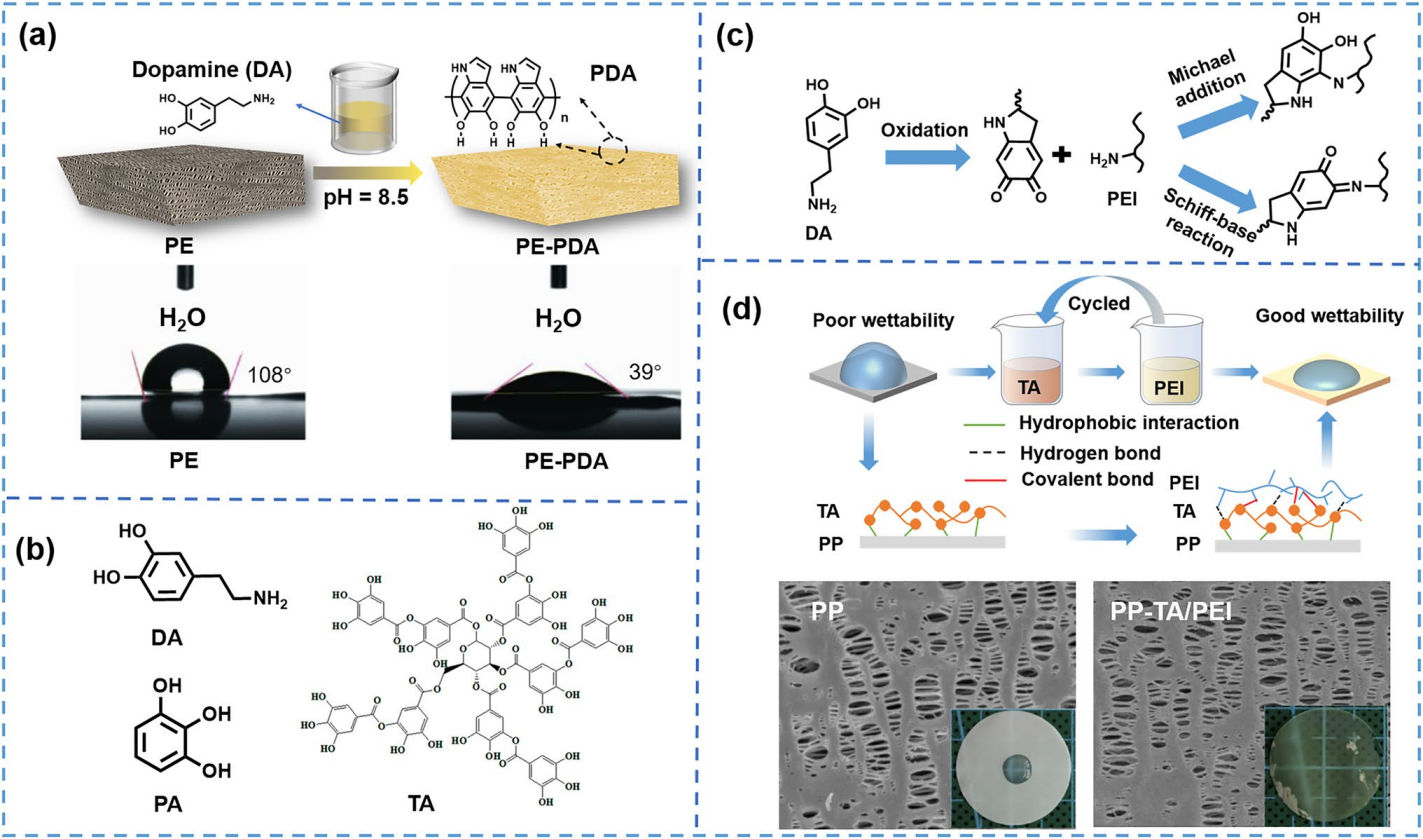
**a** Schematic illustration of the PDA on PE [27]. Copyright 2011, WILEY–VCH. **b** Structural formula of DA, PA, and TA. **c** Schematic illustration of the co-deposition of DA and PEI on PP separator [191]. Copyright 2020, WILEY–VCH. **d** Schematic illustration of the layer-by-layer self-assembly of TA and PEI [192]. Copyright 2022, Elsevier

Inspired by mussels that exhibit superior adhesion in nature, researchers found that dopamine (DA) with both catechol and amine groups can be used as the basis for strong adhesion [195–197]. For instance, Choi et al. [27] reported a kind of separator modified by polydopamine (PDA) in situ by self-polymerization of DA in the buffered aqueous solution at pH 8.5 (Fig. 10a). In this way, a very thin coating could be obtained, which would not cause pore clogging. Due to the hydrophilic nature of PDA, the wettability (the contact angle with water droplets decreasing from 108° to 39°) and the electrolyte uptake (increasing from 96% to 126%) of modified separator was significantly improved, which led to a 78% increase in ionic conductivity (from 0.23 to 0.41 mS cm^−1^). Besides, the exceptionally strong adhesion between the PDA and basement separator was beneficial for long-term battery operation. Shi et al. [187] prepared a thin PDA layer in situ on the surface of polyvinylidene fluoride-hexafluoropropylene (PVDF-HFP) nano-fibers, forming a unique core–shell structure. The modified separator had contact angle with electrolyte of almost 0° and high electrolyte uptake (254%). The ionic conductivity of modified separator was 1.40 mS cm^−1^ which was much higher than the PP separator (0.80 mS cm^−1^). Similarly, Zhou et al. [188] reported a novel separator with well electrochemical and mechanical properties by in situ modification of virgin cellulose paper (CP) with PDA, thus reducing the contact angle from 37° to 0°. Due to the sufficient hydrophilic functional groups and their affinity for electrolytes on the modified separator, it exhibited a much higher electrolyte uptake of 320 wt%. Therefore, it showed a higher ionic conductivity (1.29 mS cm^−1^) and excellent cycling stability without significant capacity decay after 600 cycles at 5C. However, the DA is not suitable for practical applications because it is too expensive. Therefore, more coating materials similar to DA were developed, such as tannic acid (TA) and pyrogallic acid (PA) (Fig. 10b), which could be directly extracted from natural materials [198, 199]. For instance, Wang et al. [190] prepared PA coating on the surface of PP separator by its in situ self-polymerization, successfully enhancing the hydrophilicity and electrochemical performance. Similarly, Pan et al. [189] coated TA on PP separator by its in situ self-polymerization in weak alkaline aqueous solution, enhancing the wettability (the contact angle with water droplets decreasing from 120° to 73°) and liquid electrolyte uptake while maintaining the original pore structure.

Although the DA or similar materials (TA, PA) have been used for modification of the separator surface and achieved good results in improving wettability, they easily form aggregates or depositions due to non-covalent interaction including hydrogen bond and π-π staking among oligomers, which causes uneven surface and inhomogeneous deposition of Li^+^ on the surface of the lithium anode [200, 201]. Besides, this process takes a long time, not conducive to large-scale application. Li et al. [189] found that the co-deposition of DA and polyethyleneimine (PEI, 600 Da) decreased the non-covalent interaction. The PP separator was soaked in Tris–HCl buffer solution (pH = 8.5, 50 mM) including polyethyleneimine and dopamine with a mass ratio of 1:1 for 1 h. This strategy not only destroyed the formation of aggregates but also formed the thinner and more uniform coating with short coating time (Fig. 10c). The contact angles with water droplets of PP, PP-DA, and PP-DA/PEI separators were 114.4°, 58.7°, and 46.3°, respectively, indicating that the introduction of PEI further improved the surface hydrophilicity due to its higher nitrogen content. The enhanced wettability brought a high electrolyte uptake, contributing to increasing ionic conductivity and capacity. Coincidentally, Zhang et al. [192] prepared a thin and uniform hydrophilic coating in situ through the layer-by-layer assembly of TA/PEI (Fig. 10d). Immersing the separator in solutions of TA and PEI in sequence, the self-assembly of TA and PEI by hydrogen bonding interaction and oxidation polymerization ensured the adhesion of the coating. In addition, it controlled over the thickness of the coating at a molecular level. The modified separator showed improved wettability with electrolyte (the contact angle decreasing from 45° to 12.7°), higher electrolyte uptake (increasing 42% compared with the PP separator), and faster penetration rate. Besides, the nitrogen atoms could break off the solvation sheath of lithium-ion to promote migration, increasing the *t*_Li_^+^ from 0.28 to 0.44.

**Fig. 11:**
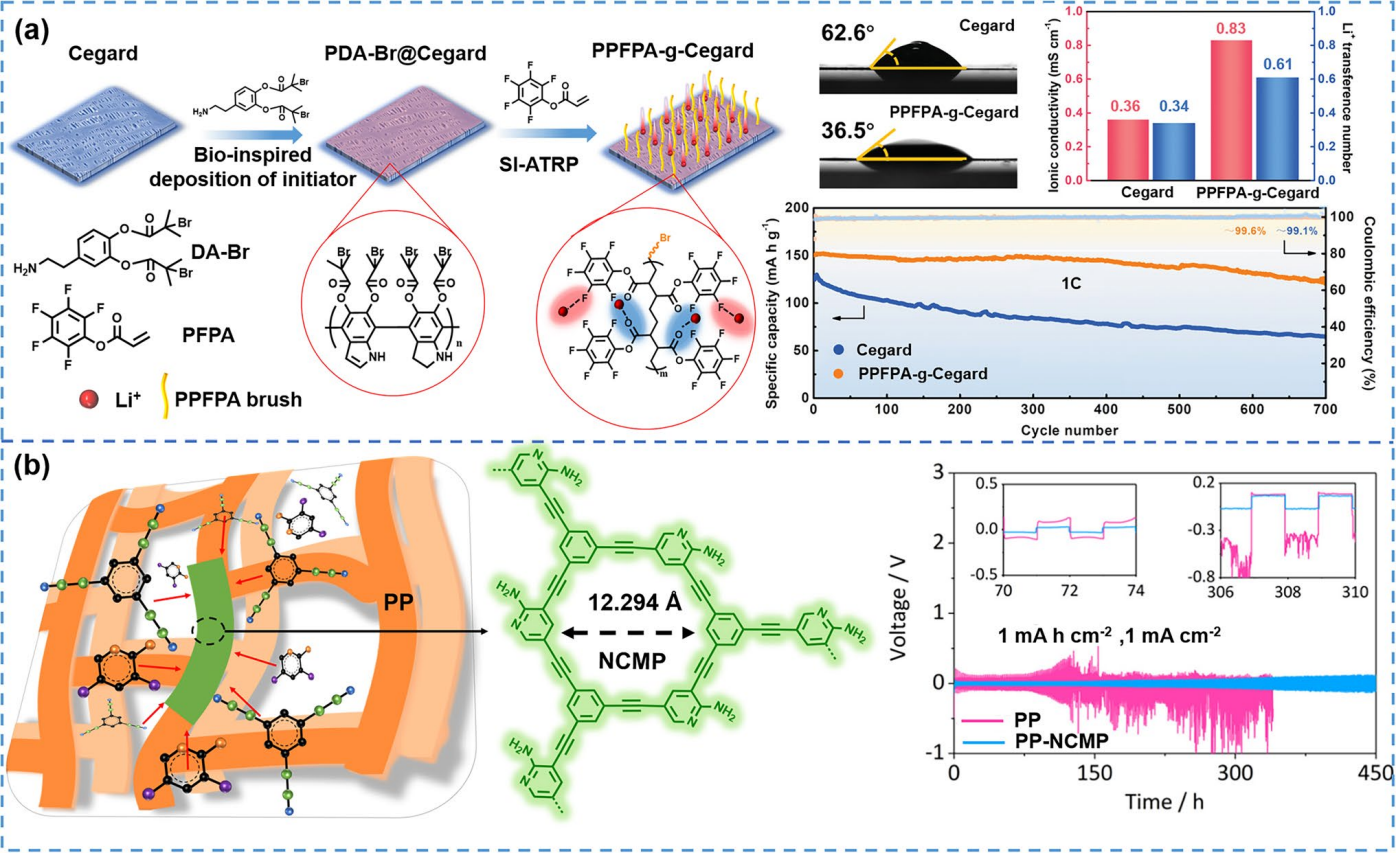
**a** Schematic illustration of PDA-assisted surface-initiated ATRP (SI-ATRP) strategy to in situ grow electronegative PPFPA polymer brushes on the Celgard separator [193]. Copyright 2020, WILEY–VCH. **b** Schematic illustrations of the NCMP in situ growth on PP [135]. Copyright 2022, American Chemical Society

DA can self-polymerize on the inert substrate surface to form PDA, and it is considered as an excellent surface modification material. Therefore, active sites can be introduced on the surface of the base separator through its self-polymerization without damaging the base separator structure. The active sites further triggered subsequent modification of the separator. Zheng et al. [193] reported an electronegative poly (pentafluorophenyl acrylate) (PPFPA) polymer brush-grafted PP separator by a PDA-assisted surface-initiated atom transfer radical polymerization (SI-ATRP) strategy (Fig. 11a). Concretely, DA containing 2-bromoisobutyryl bromide (DA-Br) was introduced to PP separator by bio-inspired self-polymerization to produce the uniform coating layer containing initiator. Afterward, the Br atom on the surface of coating induced the ATRP reaction, thus achieving high-density PPFPA polymer brushes. The polar PPFPA polymer chains contained abundant F and O atoms to improve the wettability with electrolyte (the contact angle decreasing from 62.6° to 36.5°). Besides, uniform PPFPA polymer brushes could shape highly directed 1D Li^+^ flux paths to induce rapid diffusion and uniform deposition of Li^+^. The ionic conductivity of modified separator was significantly improved (twice the ionic conductivity of PP). It thus contributed to the uniform nucleation and deposition of Li metal and effectively maintained the structural integrity of SEI in both ether and carbonate electrolytes. Moreover, the LiFePO_4_/Li half-cell assembled with the modified separator also exhibited a high discharge capacity of 150.2 mAh g^−1^ (increasing 21% compared with the PP separator) and a high-capacity retention rate of 83% after 700 cycles at 1C.

Expect organic materials like DA, conjugated microporous polymers (CMPs) can also modify separators through in-situ polymerization. Yang et al. [135] proposed a strategy of in-situ modification of polyolefin separator using an N-rich conjugated microporous polymer (NCMP), which generated ultra-thin NCMP coating on the entire outer and inner surface of PP (Fig. 11b). The NCMP coating had rich N-containing groups (-NH_2_ and -N =), uniform nanopores (12.294 Å) and π-conjugated structure, effectively suppressing LiPS shuttle, molecular sieve effects and Coulomb interactions. At the same time, the NCMP-PP separator has good electrolyte wettability (the contact angle with electrolyte < 5°) and high electrolyte absorption capacity. The nanopores of NCMP with abundant N-containing groups were larger than twice the Debye length of Li^+^ (3.0 Å), which facilitated the passage of Li^+^. Meanwhile, the highly electronegative π-electron cloud of the NCMP repelled the passage of TFSI^–^ (7.9 Å) via the Coulombic interaction. This modified separator was able to generate a uniform Li^+^ flux between the separator and the surface of the lithium metal anode, forming a stable SEI layer and smooth lithium anode without dendrite. The assembled lithium symmetric cells thus showed long-term cycling performance with a current density of 1 mA cm^−2^. Therefore, Li–S battery exhibited excellent rate performance (674 mAh g^−1^, 3C), high cycle stability (0.028% capacity degradation per cycle at 0.2C for 800 cycles), and ultra-low self-discharge (3.4% capacity degradation within 10 days).

The polymer electrolyte obtained by the separator-assisted in situ process can also be regarded as a special process of in situ modification of the separator. In this process, a less viscous precursor in the liquid state consisting of low molecular weight monomers, Li salt, initiator, or catalyst is added to the base separator and assembled into a cell, thus the precursor polymerized inside the sealed cell, which can be seen that the polymer modification coating is formed in situ on the separator without adding liquid electrolyte. This method can not only form a well contact between electrolyte and electrode but also avoid organic solvent leakage and spontaneous combustion for high [202–205]. For instance, Guo et al. [206] prepared a block copolymer electrolyte (BCPE) consisting of RAFT polymerization of poly (ethylene glycol) methyl ether acrylate (PEGA) and carboxylic acid-catalyzed ROP of caprolactone (CL), which were fabricated in situ in cellulose separator. The block structure balanced the ability of Li salt dissociation and Li^+^ coordination while reducing the crystallinity of polymer, thus showing high ionic conductivity (0.185 mS cm^−1^) and high *t*_Li_^+^ (0.59). Besides, the compatibility between electrolyte and electrode was also improved, promoting the rapid transport and uniform deposition of Li^+^. Therefore, it exhibited excellent cycling performances, with a retention rate of 92% after 400 cycles at 1C.

In summary, in situ formation of organic materials represents an environmentally friendly, efficient, straightforward, and widely adopted modification method. Organic compounds, such as dopamine (DA), are among the most prevalent organic modification materials owing to their robust self-polymerization capabilities and excellent adhesion properties. Furthermore, they possess the ability to introduce active sites conducive to additional modifications, including self-assembly and ATRP polymerization.

### Section Summary

Overall, compared with coating modification, in situ modification has the following advantages: it enhances adhesion, facilitates the creation of thinner and more uniform coatings, preserves the pore structure, and reduces interface impedance. However, its application is currently constrained by the limited availability of polymerization materials and the complexity of the processes involved.

## Grafting Modified Separators

While in situ modification has indeed effectively improved adhesion, the coating and the base separator are still primarily bound through non-covalent interactions, resulting in adhesion that is somewhat limited. Grafting modification offers a solution by enabling the formation of chemical bonds between the coating and the base separator, thereby achieving stronger adhesion while simultaneously enhancing wettability [8, 207, 208]. The characteristics and electrochemical performance of grafting modified separators by inorganic modification materials are in Table 9.

**Table 9:** The characteristics and electrochemical performance of grafting modified separators by inorganic modification materials

Separator	Coating thickness (μm)	Porosity^a^ (%)	Coating mass fraction (%)	Contact angle	Electrolyte uptake	Ionic conductivity (mS cm^–1^) at RT	Cyclic performance	References
PE/TiO_2_	–^b^	–	–	89° (H_2_O)	–	0.50	> 90% after 100 cycles at 0.2C	[209]
PE/SiO_2_	–	–	–	79° (H_2_O)	–	0.45	> 90% after 100 cycles at 0.2C	[210]
PP/FeOOH	–	–	–	19.7° (electrolyte)	–	–	80.5% after 500 cycles at 5C	[211]
PP/AA	–	–	–	75° (H_2_O)	280%	–	78% after 50 cycles at 0.2C (original) > 90% after 50 cycles at 0.2C (modified)	[212]
PP/CFP	–	–	–	26.5°(electrolyte)	–	0.99	53% after 800 cycles at 1C (original) 87.6% after 800 cycles at 1C (modified)	[213]
PP/PAM + SiO_2_	3	45	–	37° (H_2_O)	436%	1.43	–	[214]
PP/PE/PP/SiO_2_ + TEOS	0.6	–	28	–	–	0.16	91% after 65 cycles at 0.2C (original) 96% after 65 cycles at 0.2C (modified)	[215]
PP-S/PPS + CTS	0.73	–	–	7.4° (electrolyte)	–	1.01	52% after 300 cycles at 0.5C (original) 94.5% after 300 cycles at 0.5C (modified)	[216]
PE/PEI + SiO_2_	–	47.6	–	24.6° (H_2_O)	398%	0.49	79% after 100 cycles at 0.5C (original) 90.1% after 100 cycles at 0.5C (modified)	[217]
PE/PAA + ZrO_2_	–	–	–	39° (H_2_O)	325%	0.51	–	[218]

^a^ “Porosity” is the entire separator’s, instead of the coating’s. ^b^ “–” means not mentioned

### Chemical Grafting Modification

**Fig. 12:**
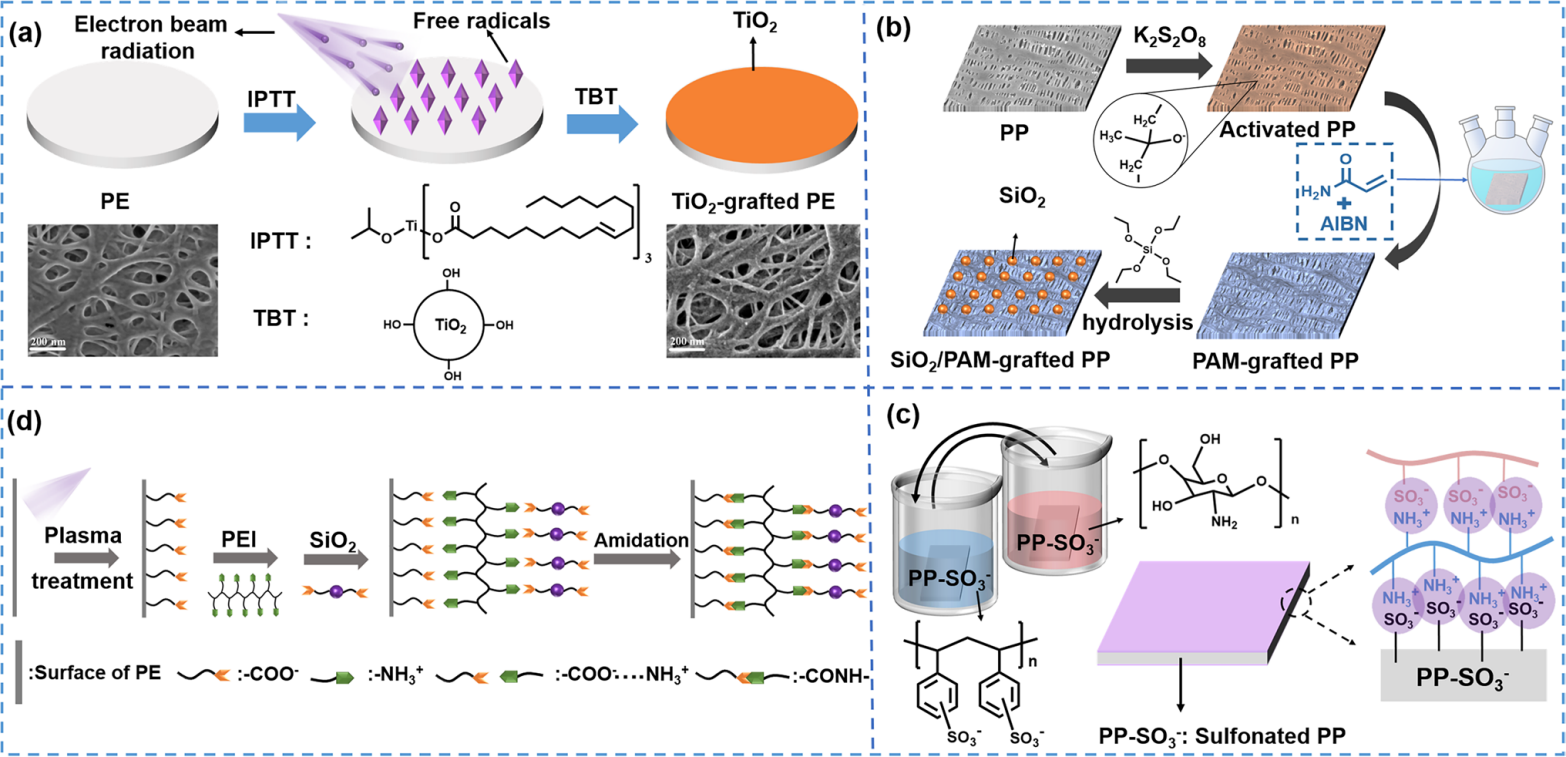
**a** Preparation process of the TiO_2_-grafted PE separator by electron beam radiation [209]. Copyright 2016, Elsevier. **b** Schematic illustration of grafting AM and SiO_2_ onto the surface of PP separator by chemical initiator decomposition [214]. Copyright 2017, Elsevier. **c** Schematic of the fabrication process of the electrostatic self-assembled functional layers on the PP separator [216]. Copyright 2023, Elsevier. **d** Schematic illustrations of the experimental process and Intermolecular interactions occurred in the process of self-assembly [217]. Copyright 2015, American Chemical Society

Chemical grafting techniques can generate active sites on the inert surface of separators through pre-treatments such as UV radiation, plasma treatment, and chemical initiator decomposition [219–223]. These active sites facilitate the formation of chemical bonds between modification materials and the separator, leading to a robust and secure binding. Additionally, the coating layers formed through these methods are extremely thin, ensuring that the pore structure of the separator remains well-preserved. [224, 225]. The inorganic modification materials (e.g., SiO_2_, TiO_2_, Al_2_O_3_) show excellent ability to improve wettability and reduce the risk of coating detachment during battery assembly and charge/discharge by the chemical grafting method. For instance, Zhu et al. [209] prepared a new TiO_2_ ceramic-grafted polyethylene (TiO_2_-grafted PE) separator by electron beam radiation (Fig. 12a). The study indicated that the TiO_2_-grafted PE separator not only showed improved wettability (the contact angle with H_2_O decreasing from 112° to 89°) and ionic conductivity (increasing from 0.32 to 0.5 mS cm^−1^), but also exhibited highly enhanced thermal stability. Besides, the thickness and pore structure of modified PE separator were similar to original PE. Except for inorganic modification materials, the organic modification materials can also significantly improve wettability by introducing polar groups (e.g., C = O, COOH, NH_2_). For instance, Yin et al. [212] grafted acrylic acid (AA) onto the surface of PP separator by an atmospheric pressure glow discharge plasma jet (APGD-PJ) while retaining the pore structure of the base separator. The introduction of AA enhanced wettability, thus reducing the contact angle with H_2_O (decreasing from 112° to 75°), and the electrolyte uptake of modified separator increased nearly 4 times compared with the original PP separator. Furthermore, the cyclic stability, discharge specific capacity, and Coulombic efficiency of LiFePO_4_/Li half-cells assembled with modified PP separators increased noticeably. Besides, the organic/inorganic hybrid strategy was utilized to enhance the thermal dimensional stability, wetting ability, and electrochemical properties of separators. As shown in Fig. 12b, Liu et al. [214] grafted acrylamide (AM) onto the PP separator by initiator decomposition, then reacted with tetraethylorthosilicate (TEOS) to obtain SiO_2_ particles and ultimately prepared a novel SiO_2_/PAM-grafted PP separator. As a result, the modified separator demonstrated superior wettability (the contact angle with H_2_O decreasing from 105° to 37°) and better thermal stability compared with the unmodified separator.

### Further Modification after Chemical Grafting

After chemically grafting active groups on the surface of the base separator, further modification is feasible. For instance, Yang et al. [216] prepared a multifunctional layered separator by grafting the SO_3_^−^ group to induce self-assembly (Fig. 12c). This study introduced the SO_3_^−^ group on the surface of the PP separator first. Then, the strong electrostatic interaction between SO_3_^−^ groups and NH_3_^+^ groups caused chitosan to tightly adhere to the surface of the separator. Similarly, polystyrene sulfonate (PSS) adhered to the surface of chitosan layer relying on the electrostatic interaction and finally formed the nanoscale self-assembled functional layers with nanoscale thickness. The grafting layers significantly decreased the contact angle from 46° to 7.4°. More importantly, the assembled Li-LiNi_0.8_Co_0.15_Al_0.05_ full cells with modified separator showed excellent cycling stability with a high-capacity retention of 94.5% after 300th cycles at 0.5C under an ultra-lean electrolyte condition of 4.8 g A h^−1^. Coincidentally, Wang et al. [217] demonstrated a self-assembly process of oppositely charged polymer polyethyleneimine (PEI) and inorganic oxide SiO_2_ for the construction of an ultrathin layer on the surface of PE separator (Fig. 12d). They treated PE separator by CO_2_-plasma to give rise to carboxyl-activated surface, and constructed PEI/SiO_2_ ultra-thin layer on the surface of carboxylated PE separator. Through the imidization between carboxyl groups and amino groups, the interlayer cross-linking happened. The electrolyte wettability of the modified separator was significantly improved without obviously increasing in separator thickness or blocking the original micropores (contact angle with H_2_O decreasing from 124° to 24.6°). Meanwhile, the modified separator demonstrated better cycle performance for working about 100 cycles with a high-capacity retention (90.1%) compared to the unmodified one (79.0%).

### Section Summary

**Fig. 13:**
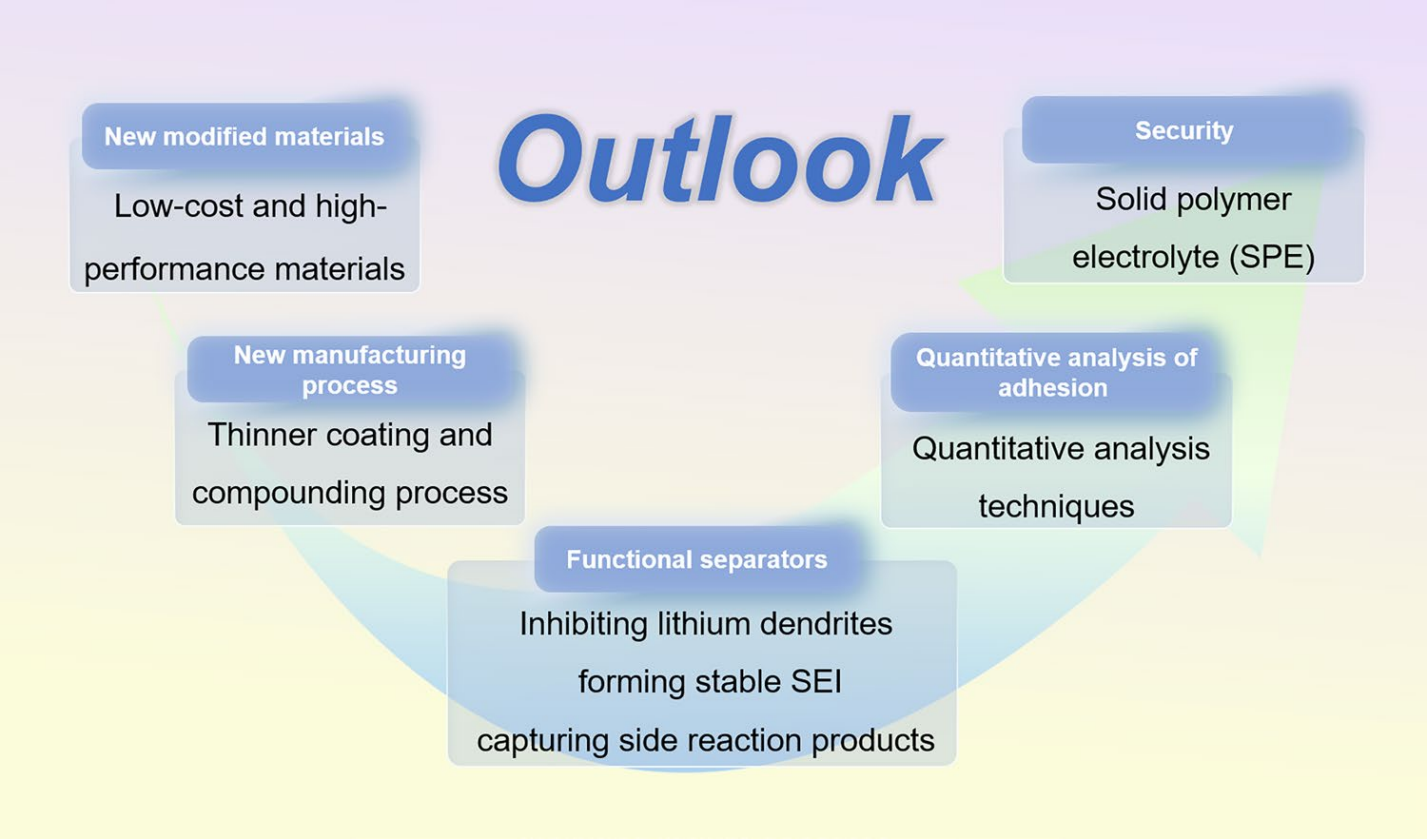
The outlook for the development direction of separators

In general, grafting modification is an effective method that not only enhances the wettability of separators but also addresses the issue of weak adhesion between the coating and the base separator. However, most pre-treatments, such as UV radiation, plasma treatment, and high-energy radiation, necessitate complex and costly equipment, making it challenging to achieve large-scale production. Excessive pre-treatment can also lead to significant damage to the porous structure, resulting in decreased ion conductivity and mechanical strength. Additionally, this damage to the pore structure causes a reduction in the porosity of the modified separator [226–230]. These limitations hinder the further application of surface modification techniques (Fig. 13).

## Conclusions and Outlook

### Conclusions

In lithium batteries, the separator plays a crucial role as it acts as a physical barrier between the positive and negative electrodes while also facilitating ion transport. Various properties of the separator, such as its thickness, pore structure, wettability, mechanical properties, and dimensional thermal stability, significantly impact the battery’s performance. Given that traditional separators cannot meet the demands of more advanced lithium batteries, the improvement of existing separators and the development of new separators are urgent priorities. Researchers have made numerous attempts to optimize traditional separators, and in this review, we have summarized the three most commonly utilized modification methods, focusing on enhancing wettability and adhesion.

Firstly, we discussed the coating modification of the separator and introduced different coating materials. Coating modification is a simple and effective method to improve electrochemical performance, and thus large-scale production has been realized in industry. However, the weak adhesion between the coating and the base separator, as well as the uncontrollable coating thickness, lead to compromised battery performance. Then, in situ modification was proposed as an alternative strategy. Enhancement of adherence is accompanied by a thinner and more uniform coating. As a result, the performance of the battery was satisfactorily improved. However, limited polymerization materials and sophisticated processes limited its application. Lastly, several chemical grafting modification strategies were listed. Although the adhesion produced by this method was the strongest among the three modification methods, it usually required complex and costly instruments, as well as destroyed the separator structure.

In summary, a detailed comparison is made of the properties, such as wettability and adhesion, of modified separators and the assembled batteries. The enhancement of wettability can be directly observed in the improved electrochemical performance. Notably, separators prepared through in situ modification exhibit superior cyclic performance compared to other methods, which may be attributed to their lower interface impedance. However, it is evident that there is a lack of quantitative analysis on the adhesion of coatings in many studies. Additionally, there are also some sophisticated modification methods that do not fall within the three categories discussed here, such as the combination of multiple different modification techniques. methods.

### Outlook

Based on the three modification methods of separator, the following future directions are proposed toward future advanced lithium batteries:

**(1) New modified materials.** At present, polyolefin separators and coated polyolefin separators (coating materials including Al_2_O_3_, SiO_2_, boehmite, PVDF, aramid fiber, etc.) are still the mostly used commercial separators. Although further improvements are expected for these separators, other modification materials usually stay in the laboratory stage due to cost. Therefore, low-cost and high-powered materials must be developed to promote the commercialization of advanced modified separators. For instance, poly(ε-caprolactone) (PCL) is a popular polymer in the field of electrolytes with stable electrochemical window (~ 5 V), high dielectric constant, weak interaction with Li^+^, low-cost and environmentally friendly features. Few studies have used PCL as coating materials to modify separators. Ye et al. [231] innovatively used blended of PCL and PEO as coating materials to modify the PP separator, significantly promoting the cycle stability of battery. New functional coating materials are expected to be developed.

**(2) New manufacturing process.** In addition to the development of new materials, the new manufacturing process also needs to be considered, which has a significant effect on coating thickness and adhesion. At present, the thickness of commercial coated separators is about 20 μm, which means large quantity of coating material and reduced energy density of the battery. Besides, the coating materials are also at risk of peeling. Therefore, developing new manufacturing processes to decrease coating thickness and improve adhesion is conducive to the development of advanced lithium batteries. In the laboratory, researchers are no longer limited to single modification methods, but prefer the combination of multiple modification approaches. It can be predicted that the combination of multiple methods and materials may be the development trend.

**(3) Functional separators.** In addition to the basic role of the separator in transporting ions and preventing short circuits, it is also expected to acquire other functions such as inhibiting lithium dendrites, forming stable SEI and capturing side reaction products. The non-uniform lithium flux in lithium metal batteries is the main reason for the growth of lithium dendrites. Therefore, the separator with the function of promoting the uniform distribution of Li^+^ can effectively inhibit the growth of lithium dendrites. Materials with uniform nanochannels, including COF, MOF, brush polymer, inorganic and organic porous materials, contribute to the uniform distribution of Li^+^, and thus they are used as modified materials to endow the separators with lithium dendrites inhibition ability. Besides, imparting the separator with cation selectivity is also an innovative strategy, which greatly promotes the migration of Li^+^ and inhibits anion diffusion, achieving smooth lithium deposition [232–234]. The side reaction between lithium metal and electrolyte results in the formation of SEI. However, unstable SEI would cause the unreacted lithium metal to be exposed to the electrolyte, resulting in continuous decomposition of the electrolyte and ultimately a decrease in battery life. Functional separator can be designed to construct a stable SEI by sustained-release strategy and form artificial SEI. Traditional separators can be used as substrates to release active substances such as inorganic salt and transition metal oxides, which slowly dissolve in the electrolyte during the cycle and react with lithium metal to form stable SEI. Another strategy is to prepare reactive separators with the function of constructing artificial SEI. In this method, the coating on the surface of the separator that can react with lithium metal, such as organic (lignosulfonate) and inorganic salt (boehmite, SnS_2_). This in situ artificial SEI layer can protect the lithium anode from electrolyte side reactions and inhibit the degradation of liquid electrolytes. In the process of charge and discharge, some by-products are inevitably produced (HF, transition metal ion, polysulfide), which shuttle in the battery causing capacity attenuation and battery life decline. They can be suppressed by introducing specific groups or coatings on the separator surface to inhibit or capture these products.

**(4) Quantitative analysis of adhesion.** Adhesion of coating is an important parameter in industry that deeply affects the safety and cycle stability of batteries. The coating with poor adhesion may fall off from the separator, generating cracks between the separator and the electrodes, thus resulting in increased internal impedance of the battery, uneven current density distribution, inhomogeneous deposition of lithium and lithium dendrite growth, seriously affecting the performance of the battery. At present, few researchers have conducted quantitative analysis of adhesion, and most of them use qualitative methods, such as observing whether the coating falls off by folding and bending the separator. Therefore, we expect more studies to characterize adhesion through quantitative analysis techniques (such as 180° peel test).

**(5) Security.** With the rapid development of high-energy density batteries, safety issues have become one of the great challenges to be solved. Flammable organic solvents in liquid electrolytes are one of the main factors affecting the safety of batteries. Solid polymer electrolyte (SPE) is considered as a good solution strategy due to the absence of traditional organic solvents. In particular, the polymer electrolyte is prepared by the separator-assisted in situ process to effectively enhance the efficient Li^+^ transport between anodes and cathodes [235]. Although there is a gap in electrochemical performance between the SPE and liquid electrolyte, SPE is considered as a promising development direction for high-safety lithium-ion batteries in the future.
